# An Open‐Source Systematic Reviews Integrated System (OSSYRIS) – Streamlining Processes and Standardising Data Structures

**DOI:** 10.1002/cesm.70088

**Published:** 2026-06-05

**Authors:** Xavier Bosch‐Capblanch, Christian Auer, A. S. M. Sayem, Guillaume Deschamps, Salvador Camacho, Luís Segura, Salem Al‐Aidroos, George Tsey Sabblah, Kaspar Wyss

**Affiliations:** ^1^ Swiss Tropical and Public Health Institute Allschwil Switzerland; ^2^ University of Basel Basel Switzerland; ^3^ Freelance consultant Little Rock Arkansas USA; ^4^ Food and Drugs Authority (FDA) Accra Ghana

**Keywords:** data extraction, integration, open‐source, systematic reviews

## Abstract

**Introduction:**

Carrying out a systematic review (SR) of the literature entails a high workload and encompasses a variety of very different tasks. The emergence of artificial intelligence tools has brought further opportunities to improve the efficiency and reliability of SRs. SR processes can be optimised to the extent that integration and interoperability of software tools across production stages are progressively implemented. A key stage is data extraction, which can be challenging due to the large amounts of data items to consider and the variability of studies reporting styles, which heavily complicates data processing and analyses.

We report the development of a software platform that integrates processes across all types of SR tasks, including overviews of SRs, is open source, and addresses the challenges of data extraction through the standardisation of data structures: the “Open‐Source SYstematic Reviews Integrated System” (OSSYRIS).

**Methods:**

We established a series of criteria to select the software integrated in OSSYRIS: few applications, covering all SRs production processes, inter‐operable and open source. After several trials, we selected Zotero as reference manager, KoboToolbox XLSForms for screening and data extraction and R for analyses and reporting. We integrated all components using Application Programming Interfaces (API) in R. For the data extraction form, we identified content items from our own experience and from the Cochrane handbook. OSSYRIS has been piloted and used in several SRs and overviews carried out by the authors.

**Results:**

In OSSYRIS, references are manually imported in Zotero and are integrated into XLSForms in KoboToolbox, which are used for online screening by reviewers. R automatically downloads the screening results from KoboToolbox and updates the status of the references in Zotero as ‘irrelevant’, ‘included’, ‘excluded,’ and ‘unclear’. R automatically produces the figure with the PRISMA flow of studies and references lists by status, for reporting.

Data extraction is manually done using another XLSForm structured in sections: study characteristics, participants, intervention or exposure, outcomes, results and conclusion. Data extraction is standardised by using pre‐coded data items, filtering data items according to relevance criteria and modularising data structures. Results of studies are entered using a data structure consistent with the information on the type of outcomes, in a form preceding section. Items that require a decision based on certain criteria, such as which is the type of study or the risk of bias assessments, are not filled in by reviewers; rather reviewers enter the criteria and OSSYRIS internal algorithms issue the specific type of study design or the risk of bias assessments, based on those criteria. XLSForms provide additional functionalities to ensure data integrity. R automatically produces the characteristics of included studies and other analytical outputs for reporting. Standardisation and modularity facilitate adapting the form for different types of SR.

**Conclusions:**

OSSYRIS provides an open source, integrated system to carry out SRs. Our work may support the promotion of open source and free tools to conduct SRs bringing together a community of practice to further improve it, within Cochrane and beyond.

## 1 Introduction

Carrying out a systematic review (SR) of the literature entails a high workload and encompasses a variety of different tasks, from protocol development, through literature searching, screening and data extraction of studies, up to analyses and reporting. There are substantial efficiency and effectiveness gains across these tasks by using appropriate software [1]. The emergence and development of artificial intelligence (AI) tools have brought further opportunities to improve the efficiency and reliability of SRs, not without some risks, such as compromising evidence synthesis standards or the proliferation of tools which are not properly evaluated [2]. AI tools have created tangible efficiency gains across SR workflows, particularly in titles and abstracts screening [3], PICO (Participants, Intervention, Comparison and Outcome) identification and in some components of data extraction, particularly from randomised controlled trials (RCT) [2]. For example, recent evaluations show that large language models (LLM) assisted screening can achieve acceptable sensitivity and specificity with reduced reviewers' time use [4]. However, end‐to‐end automation is not feasible yet and, accordingly, AI should be implemented as a human‐in‐the‐loop aid, complying with AI implementation standards, as they are developed [5].

SR processes are increasingly optimised as integration and interoperability across production stages are progressively considered. For example, covidence [6] can import Research Information System (RIS) files and its extracted data can be further exported to be read in RevMan [7] for analyses and reporting. Most of the software tools, though, are proprietary and have costs.

A key stage is data extraction (including risk of bias (ROB) assessments), because it produces the data that will become the body of evidence in SRs. Data extraction is challenging because studies in a SR may differ in (i) the terminology used (e.g., for study designs), (ii) the detail with which study aspects are reported, (iii) data formats and (iv) analytical approaches. Besides, data extraction must comply with reporting requirements, such as the PRISMA checklist [8], which counts with more than 15 extensions [9]. SRs outside the clinical domain (e.g., in health systems) further challenge data extraction, with a larger variety of study designs, having non‐human entities as participants (e.g., health facilities), with multifaceted interventions and many different types of outcomes. Some of the most commonly used tools have limited control on data structures and flows (e.g., covidence [10]) or have, in our view, a rather steep learning curve (e.g., EPPI‐Reviewer [11]), with limited AI integration [12].

To our knowledge, there is not a single software platform that simultaneously (i) integrates processes across all SR tasks, (ii) is open source and free, (iii) addresses the challenges of data extraction and (iv) provides a standardised data structure. We report in this paper the development and functionalities of such a platform, designated as “Open‐Source SYstematic Reviews Integrated System” (OSSYRIS).

## 2 Methods

The criteria to select the software to build OSSYRIS [13] were: (i) to use as few different applications as possible; (ii) covering as many stages of SRs production as possible, including management of literature references, screening for relevance based on titles and abstracts, full texts inclusion and exclusion, data extraction, analyses and reporting; (iii) inter‐operability among them based on the availability of Application Programme Interfaces (API); and (iv) open‐source.

Recognising the increasing importance of open‐source software in science globally [14, 15], and its growing demand [16], we scrutinised open‐source software packages that complied with the criteria described above. We also considered the data collection tools section in the Cochrane handbook [17], we searched in the Cochrane website [18] and consulted literature review tools [19].

We followed an opportunistic approach and we prioritised software packages that, complying with those criteria, were readily available to the team and entailed limited additional efforts to implement. We considered references managers (e.g., Mendeley, Zotero), statistical packages (e.g., R, EpiInfo), spreadsheets (e.g., Google sheets), forms designers (e.g., Google forms, EpiInfo, XLSForms), and generic high‐level programming languages (e.g., Python). We empirically tested their data handling capacities (e.g., number of references, cloud storage capacities), connectivity using APIs and other functionalities (e.g., branching in forms). This allowed the team to start OSSYRIS with some reassurance that we could develop a functional version of it and at the same time complete actual SR tasks in a timely manner.

As a result, we selected the following tools, for the different SR production stages:

1. Literature references management: Zotero [20] because it is an “open‐source and developed by an independent, nonprofit organization” [21] and can be accessed through APIs;
2. Screening for relevance (i.e., titles and abstracts), inclusion/exclusion based on full text, and data extraction: XLSForms [22], a tool for authoring forms in Excel, using human‐readable format. Forms are created in a workbook using XLSForms structure, syntax and terminology, then they are converted to ODK [23], KoboToolbox [24] or other XLSForms interpreters, and made available online via a web interface or an Android data collection app. We used KoboToolbox because it is free below a certain level of use and can be accessed through APIs [25].
3. Analyses and reporting: R [26], because it is open‐source, it carries out statistical analyses, produces all sorts of analytical outputs, including graphs, and can populate reports using markdown [27], QUARTO [28], or OfficeR [29]. We used R as the core of OSSYRIS because it has the APIs that can connect with the other tools: Zotero and XLSForms.

We placed special emphasis on the data extraction stage because of the challenges commonly experienced in the past, such as the violations of data structure integrity in spreadsheets, even when protected, the lack of standardisation in data descriptors or the limited use of data validation rules; all of which heavily overburden later tasks, such as data cleaning, comparisons, and analyses. For the data extraction form, we identified content items from our previous experience in carrying out SRs, from the Cochrane handbook [30] and from other tools.

OSSYRIS is designed to handle any type of SR, including overviews of SRs. The initial testing of the platform was carried out in a SR related to the implementation issues of RTS, S malaria vaccine introduction [31]. Between 2023 and 2025, we designed and programmed OSSYRIS new functionalities, and we used it in SR and overviews on DTPCV booster vaccination and rabies vaccine integration strategies, for Gavi. We considered suggestions from reviewers and debugged issues, as they appeared. In the process of building OSSYRIS, the team discovered additional features of XLSForms and R that could implement more complex functionalities.

## 3 Results

We report here (i) the overall setup and functionalities of OSSYRIS and (ii) the details of the data extraction form. We have omitted the descriptions of the screening forms (see Supporting Information: S2 and S3), analyses and reporting tasks due to space limitations and because they are relatively less challenging in terms of SR production.

### 3.1 Overview of the Platform for Screening and Data Extraction

**Figure 1:**
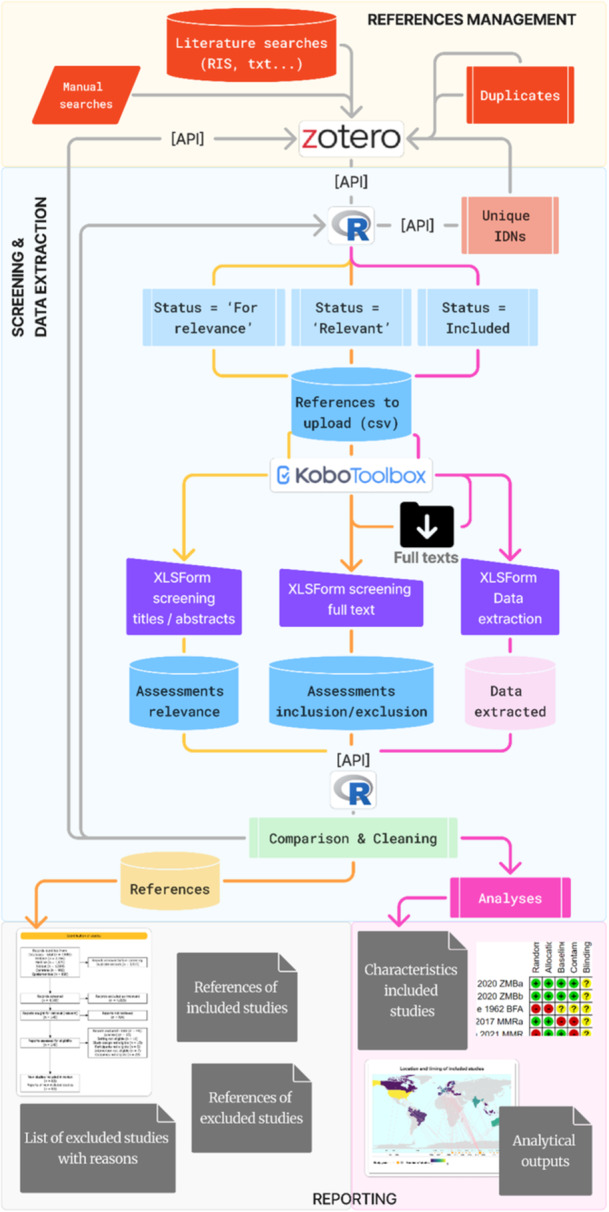
The Open‐Source SYstematic Reviews Integrated System (OSSYRIS). *Note:* colours are indicative of the continuity of processes.

Figure 1 represents the components and functionality of OSSYRIS. The platform consists of three software tools: (i) a reference manager (Zotero), which contains the references resulting from the implementation of the search strategy; (ii) data forms (XLSForms built in standard spreadsheets, converted into Extensible Markup Language (XML) and deployed in KoboToolbox) and (iii) the analytical and reporting software, also ensuring interoperability (R). The full details of the workflow can be found in Supporting Information: S1. Examples of screening forms can be found in Supporting Information: S2 (relevance of titles and abstracts) and S3 (inclusion based on full text), and an online [32]; and an example of data extraction form can be found in Supporting Information: S4 and online [33].

**Figure 2:**
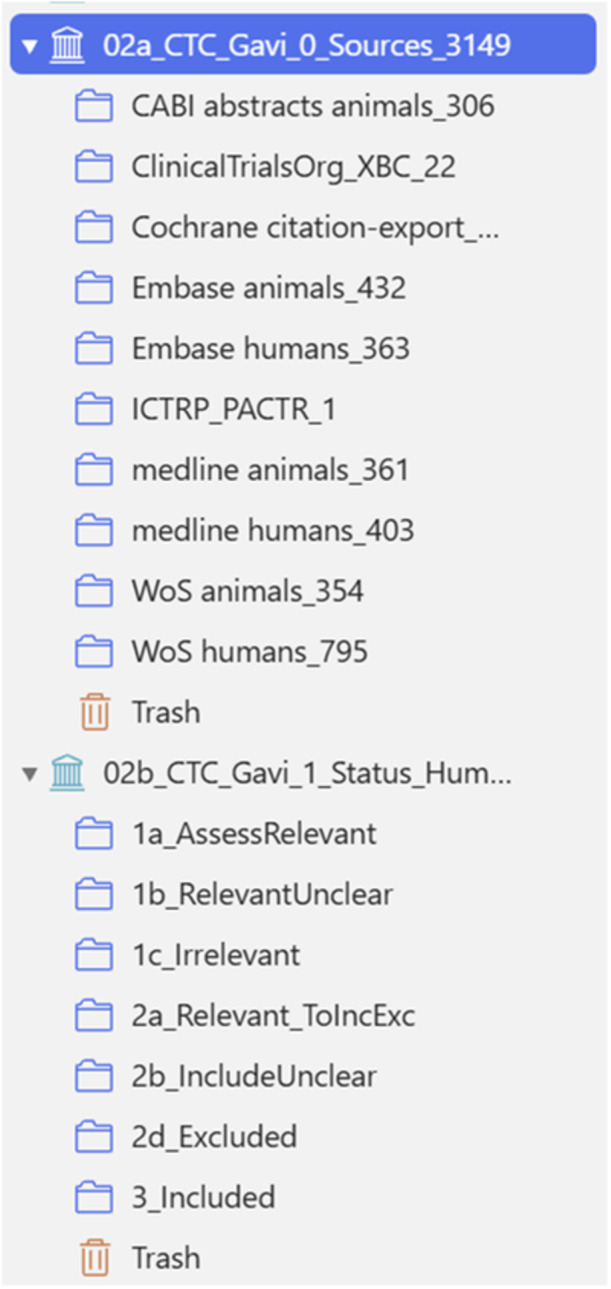
Example of Zotero collections of references, by source (with number of documents) and by status.

At the beginning of the process, references in Zotero are organised in collections specific to each literature source. To keep data integrity across the whole life of the SR production, the first task is to assign unique identification numbers (IDN) to each reference. Since Zotero does not do that automatically, R downloads the Zotero collections, produces IDNs, writes them in the field named ‘Extra’ and updates Zotero collections uploading the ‘Extra’ fields, using the corresponding API. Then, batch deduplication is run [34] in Zotero and the deduplicated references are assigned to a Zotero collection named “Assess relevance” (i.e., to screen based on titles and abstracts). During the whole life of a SR each reference is assigned a status (e.g., ‘relevant’, ‘included', ‘excluded’) that is fully consistent across platforms (see Figure 2).

OSSYRIS operates in cycles: first, references are uploaded to KoboToolbox [35] for screening; reviewers assess the relevance of titles and abstracts using the screening XLSForm; R downloads reviewers' assessments, updates Zotero's status collections via API and simultaneously produces a csv file of references with updated status that is uploaded back to KoboToolbox for full text screening; then reviewers assess references for inclusion based on full texts using the screening XLSForm; R downloads reviewers’ assessments, updates Zotero's status collections via API and simultaneously produces a csv file of references with updated status that is uploaded back to KoboToolbox for data extraction; finally, reviewers extract data using the data extraction XLSForm, R downloads the data and produces reporting and analytical outputs. In each cycle, XLSForms automatically filter the references with the appropriate status (e.g., ‘For relevance’ status for titles and abstracts screening, ‘Relevant’ status for full text inclusion/exclusion screening and ‘Included’ status for data extraction).

Reviewers carry out the screening and data extraction tasks online, via a web browser (e.g., ENKETO [36]) or the KoboCollect Android app [37], which are XLSForm implementers. Online XLSForms are organised in pages and have the following utilities for reviewers: the narrative of questions and pre‐coded options (e.g., ‘age of participants’), where applicable, hints (e.g., ‘you can only select one option’) and external links to the folder with the full text of studies and to other resources (e.g., lists of countries).

Full texts may have been produced and downloaded in Zotero; but in any case, the screening XLSForm has an initial section to check whether the full text has been uploaded or, alternatively, provides a Digital Object Identifier (DOI) or a generic search link to retrieve the full text, if available. Full texts are stored in a shared, online folder, the link to which appears in the same very form. Files are renamed to keep a standard naming convention, using the assigned IDNs. The same folder is accessed for data extraction from full texts.

The cycles described above can be implemented at any time as work progresses (e.g. when a reviewer has finished a batch of tasks) producing preliminary or final outputs in R: (i) updated Zotero collections according to the status of the references (i.e. included, excluded, unclear, relevant, irrelevant), (ii) progress monitoring of the specific reviewer tasks, (iii) comparisons of double, independently generated data, (iv) PRISMA figure of the flow of studies, (v) list of included references and (vi) of excluded references, (vii) table of excluded studies and reasons for exclusion, (viii) characteristics of included studies table, (ix) other generic descriptions of the included studies, (x) ROBs or AMSTAR graphs and (xi) a report in MS Word with all those elements in it.

### 3.2 The Data Extraction XLSForm

The data extraction XLSForm is a component of OSSYRIS. We structured the data extraction form [13] (Supporting Information: S4) into the following sections: (1a) form introduction, (1b) selection of the study and characteristics, (2) participants, (3) intervention or exposure, (4) outcomes, (5) results, and (6) conclusion. The form contains also data items for ROB specific tools in the corresponding section (e.g., if RCT, allocation concealment is in section 1b and selective reporting in section 4), as well as applicable GRADE items (e.g., indirectness related to participants or the importance of the outcomes; see Supporting Information: S4, sections 3.A.3 and 4.1.A, respectively). The features of the data extraction XLSForm are listed in Table 1.

**Table 1:** Features of data extraction XLSForms.

Domains	Feature	Comments
**Open source**		
Collaboration	All tools are open source and free	OSSYRIS is a platform that can be improved based on the whole potential of open source collaborations
**Integration**		
Interoperability	Data in R, XLSForms and Zotero remain consistent and updated all the time	R reads, updates, and controls contents in KoboToolbox, Zotero and R itself
Adaptability	Immediate adaptation as new versions of software or review components emerge	For example: new ROB tools can easily be integrated by modifying an existing ‘module’ or by creating a new module. Modules can be harboured in the XLSForm and shown or hidden according to the values of preceeding fields
**Standardisation – generic template**		
Uniformity	The same variable names and labels in XLSForms are used at all stages: from screening to data extraction, analyses and reporting	Adaptations to different SR are operated through the list of codes
Flexibility	Data items can be filtered or hidden depending on preceeding responses	Adaptations to different SRs do not need editing the individual items in the form
Modularity	Sections are independent from each other and content modules can be hidden or shown as needed	Adaptations to different SRs do not need to add or remove sections in the form
**Data control and flow**		
Data hierarchy	Variable data are automatically read from external sources data in XLSForms	This improves standardisation across SRs (e.g., list of countries, references to screen, reviewers' data)
Management	Control of users' activity with time recordings	Using the XLSForms audit function
**Data integrity**		
Unique identifiers	Unique identification numbers for each reference at the start and used across the whole platform up to reporting	Loading references to KoboToolbox server and deploying the form have to be done manually; however, it may be possible to automatically do it with appropriate APIs
Control of changes	Submitted forms cannot be manipulated without providing credentials	Reviewers cannot edit a submitted form without providing credentials; while this can be seen as an inconvenience, it guarantees data integrity
Checks	The internal consistency of XLSForms is checked before deployment	For example, duplicate variable names, errors in ‘calculation’ fields
**Data quality entry processes**		
Data formats	Multiple types of questions available	Numerical, string, dates, rank, media, etc. This allows to present rich text (e.g., form or technical help) and images, among other formats
Data validation	For numerical and character fields	It includes ‘regular expressions’, which ensures consistency of data entry
Automated decision‐making	Calculation fields for handling questions that rely on the fulfilment of certain criteria	Minimises errors when making decisions (e.g., study designs, ROB)
Pre‐coded questions	Pre‐coded or numerical fields; free text fields to provide labels, details or additional information	Most of data entry rely on pre‐coded lists or quantitative data. Allows automated data presentation and analyses across systematic reviews
Pre‐coded filters	Pre‐coded choices can be filtered	Brings consistency and avoids unnecessary burden of pre‐coded questions when items are not applicable
Structured extraction of results	Strictly structured based on entries in ‘participants’, ‘intervention’ and ‘exposure’ and ‘outcomes’	Forces reviewers to extract a clear study structure, which facilitates the understanding of study features at analytical time
**Analytical processes**		
Analytical capacity	Relies on a vast amount of R libraries	Includes string, numeric and objects manipulations; graphs as well as maps; meta‐analyses and many other types of analyses; and reporting.
Standardisation of analyses	Standard analytical code template	Possible because variable names do not change across SRs
**Reviewer usability**		
Language	Seamless change of language without additional programming or forms	Multi‐forms language [38]. However, the current version of data extraction is only in English
Help	Contents to assist reviewers in short or long texts and links	Instructions about how to fill the form; guides on how to fill in ROB, incorporated in the form itself
User‐friendliness	Certain capacity to adapt font‐sizes, colours, paging and distribution of items in the screen [39]	There are some aesthetic elements programmable in the web‐based and Android versions, but the possibilities are limited
Multi‐platform	Web‐based and mobile based	Web‐based through Enketo [36] and Kobocollect [37]

An XLSForm is a workbook with three sheets: ‘settings’, ‘survey’ and ‘choices’. ‘settings’ contains the overall parameters of the XLSForm (e.g., form name, overall format); ‘survey’ contains the form itself, with the narrative of the items to collect (e.g., ‘how many participants were enrolled?’), variables names, variables types (e.g. multiple choice, numeric, text) and a series of columns that provide a great variety of functionalities, such as data validation, filtering or calculations (see the XLSForm specifications [40] for a description of the whole spectrum of XLSForms functionalities); and the ‘choices’ sheet that contains the list of pre‐coded items to be used in single‐ or multiple‐choice questions in 'survey'.

We have created three mechanisms to standardise data. First, we have created the ‘survey’ sheet in a way that all data items are the same across all SRs and the only adaptations needed are in the ‘choices’ of six specific questions, shown in cyan colour in Supporting Information: S4. An example of the latter is the pre‐coded question about ‘Participants’, which may include pre‐coded items such as ‘new‐borns’, ‘infants’ or ‘children’ for a SR on paediatrics and ‘non pregnant’, ‘pregnant’ and ‘puerperal’ for a SR on obstetrics, stored in a list of cells named ‘P_type’ in ‘choices’. The sheet ‘survey’ containing the form, which points at a pre‐coded list ‘P_type’, does not need to be changed; only the items in that list ‘P_type’ with the pre‐coded types of participants is changed, depending on the specific SR.

**Figure 3:**
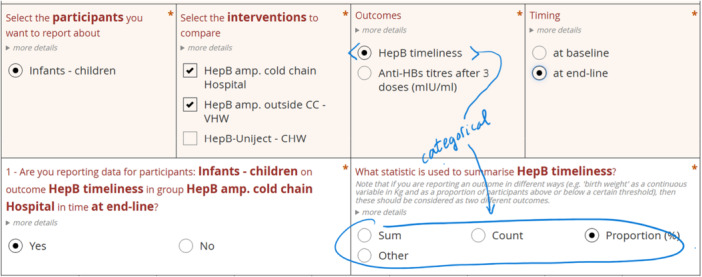
Screen shot of the Results area in the OSSYRIS data extraction form (partial view).

Second, items that would need adaption are handled with automatic filters. For example, section 5 with ‘Results’ will ask for ‘percentages’ and ‘counts’ only for outcomes classified as ‘categorical’ in section 4, where outcomes are described; and will ask for ‘means’ and ‘medians’, only for the outcomes classified as continuous or discrete (see Figure 3).

Third, further standardisation can be achieved by the modularisation of data in situations where the same data structure needs to be repeated several times. For example, when describing several outcomes, we need the same descriptors for each outcome, such as a label, the type of data used to assess the outcome or how was the outcome assessed. We have created a single module with those descriptors that can be repeated the number of times that reviewers establish at the time entering data for each particular study: a study may report on one, two or more outcomes of interest, which are the times the same module will be repeated [41]. These modules also exist in the data extraction form for the types of participants, for interventions, exposures or comparators, and for the types of results to report.

As suggested, the results section of the XLSForm to extract the findings of the included studies is modularised using the repeat functionality to extract as many findings as needed. For each finding, the module retrieves the list of participants, interventions or exposures and outcomes entered in the previous sections, which are selected by the reviewer, as applicable. The form selectively lists the statistics to report the results (see Figure 3). The form has also a free‐text field to enter a narrative for qualitative outcomes.

The high degree of standardisation achieved with the approaches described above allow to build analytical code in R with minimal if any adaptations needed across SR, because the names of the variables and their descriptors are always the same. In this way, items such as the characteristics of included studies table can be produced writing the code only once. Standardisation also facilitates the comparison of data extracted by different reviewers from the same study.

An important functionality in OSSYRIS is the ‘automation of decisions’: certain data items or questions require the judgement of reviewers based on the responses to a series of questions. For example, a study is classified as an RCT if (i) there are two or more groups that (ii) are randomly allocated to different interventions. Instead of asking reviewers ‘what is the study design?’ and presenting a series of options including ‘RCT’, the form asks about the number of groups and about how where they allocated, and then an XLSForm internal algorithm automatically assigns the corresponding study design (e.g., RCT, or controlled before‐and‐after or any other). This is done using the ‘calculation’ utility in XLSForms.

**Figure 4:**
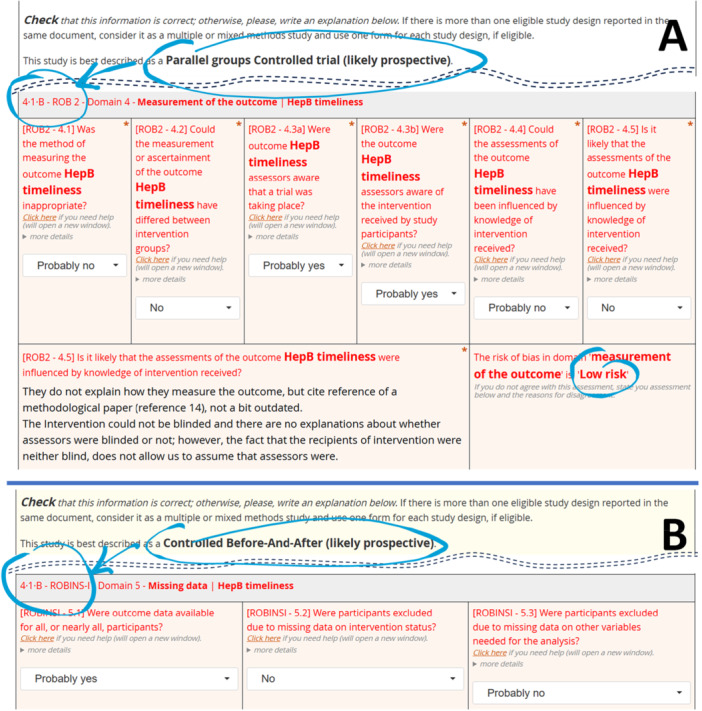
This figure shows two screen shots of non‐contiguous areas of the data extraction forms in two different systematic reviews (SR): A and B. In SR A, the study design is a randomised controlled trial, and OSSYRIS automatically shows the risk of bias tool RoB 2, later on in the form; in SR B, the study design is a controlled before‐and‐after study, for which OSSYRIS automatically shows ROBINS‐I, and hides RoB 2, later on in the form. Note as well that the RoB 2 assessment (‘Low risk’, circled in blue) is not entered by the reviewer, but it is rather the result of applying a coded algorithm that interprets reviewers’ responses to questions ROB2‐4.1 to 4.5, shown. Also, the study designs (circles in blue in A and in B) are the result of applying a coded algorithm that interprets reviewers’ responses to a series of questions about the study designs (not shown in these screen shots, but visible in Supporting Information: S4).

The calculation functionality is applied to more than 150 items in the data extraction form structure, both for producing information items and for internal checks. Another more sophisticated example is the decision‐making algorithm related to the RoB 2 tool [42]. Domain 4 in RoB 2 (i.e., ROB in the measurement of outcomes) has eight sub‐criteria and 10 decision pathways. Our form does not ask reviewers what is the final assessment of RoB 2 domain 4; instead, the form requests reviewers to complete the eight sub‐criteria (e.g. method to measure the outcome, or outcome assessors blind to the intervention) and then uses a ‘calculation’ field with an internal algorithm to establish the final assessment of domain 4 (i.e., ‘low risk’, ‘some concerns’, or ‘high risk’, see Figure 4A). Additionally, the relevance of some RoB 2 questions depends on previous RoB 2 responses, which the form filters, as described above (this is why RoB 2 stops at question 4.5 in Figure 4A; the other questions are not relevant). The form also provides the possibility to overrule the automatic conclusion issued by the form if needed, with space to write an explanation (see below ‘Low risk’ in Figure 4A). Relevant ROB issues can be found in several sections in the XLSForm, as appropriate (e.g., allocation sequences in the study design section or measurement of outcomes in the outcomes section; see Supporting Information: S4, sections 1b(C) and 4·1·B, respectively).

XLSForms provide many other features, such as a large variety of ways to enter data (e.g., text, pre‐coded, visual scales or even images). Data integrity is further ensured by using constraints to data entry, which can be numerical (e.g., blocking ages below zero) and strings using regular expressions [43]. Forms also allow to control for specific items to make them compulsory to fill in or not, before submission. Finally, XLSForms can automatically create audit data [44] that provides internal parameters related to the completion of the forms.

What would it take to adapt the data extraction XLSForm (or equally, a screening form)? (i) Structural adaptations (e.g., adding data items) are done editing the XLSForm ‘survey’ sheet, which contains the specific ‘questions’ to extract data about. For common items across SR (e.g., ‘Participants’), this should not be necessary. For other items (e.g., RoB 2, or Risk Of Bias of Non‐Randomised Studies of Interventions ‐ROBINS‐I, see Figure 4B) this can be done modularly with automatic de/activation (see Figure 4). These adaptations require familiarity with XLSForms syntax. As OSSYRIS is further developed by users, other modules with conditional activation can be seamlessly incorporated (e.g. TIDieR for reviews of interventions) and coded only once; (ii) pre‐coded content adaptations (e.g. adding ‘adolescents’ to the list of age categories in ‘Participants’) is implemented editing, adding or removing rows in the ‘choices’ sheet, which only requires basic spreadsheets literacy; and (iii) because XLSForm are open source, all features and functionalities can be customised (e.g., variables names, labels, languages, help text, links). Furthermore, XLSForms are built using standard spreadsheets tools, and variables names and labels are deployed using formulas in a way that changes in variables names or labels can be implemented straightaway based on users’ preferences without compromising the functionalities of the form.

## 4 Discussion

We have produced an open‐source and integrated platform to streamline all tasks related to the production of SRs (OSSYRIS), compliant with the FAIR data principles [45], to provide a functional platform that can be constantly improved by the large open source and SRs communities, with the current or additional tools, including AI.

Although OSSYRIS is not the first time XLSForms are used in SRs [46], it may be the first implementation of XLSForms within an integrated software environment, able to handle different types of SRs and covering all stages of SRs production. All three applications used (i.e., R, Zotero, and XLSForms) are free and they are continuously updated through open‐source contributors.

OSSYRIS forms are highly standardised allowing adaptations with minimal effort, as we have experienced using the same forms across several SRs. A generic and fully structured data extraction form also brings standardisation to dependent tasks, such as data management, data cleaning, analyses and reporting. The modularity of OSSYRIS forms facilitates both the standardisation and further adaptations of the tools to changing standards and norms in SR production.

There are many tools that can bring efficiency [1] in several stages of SRs productions. However, the evaluation of data extraction approaches should take into account the quality of extracted data [47] and the impact on data management, cleaning, analysis, and reporting processes, as well. We did not estimate the efficiency of OSSYRIS data extraction. Anecdotal observations, though, suggest that data extraction was experienced as heavier by some reviewers. However, we have also experienced efficiency gains; for example, we have used the same R code to produce the table of characteristics of included studies, as well as other analytical outputs (except for meta‐analyses that are highly SR specific) across different SR, with minimal if any additional coding.

Currently, the Methodological Expectations of Cochrane Intervention Reviews (MECIR) document does not seem to establish specific quality standards for the extracted data or for data extraction tools, although it indicates the need to use piloted data extraction forms among other features [48]. One aspect mentioned is the need to have reviewers familiarised with the data extraction form. We acknowledge that new users of OSSYRIS will have to invest some initial efforts in familiarising themselves with the forms and their functionality. Prospective users will have to assess whether the structured data and functionalities of OSSYRIS pay‐off, which may be the case particularly in SRs of diverse study designs and complex topics. The fact that the OSSYRIS data extraction form is standardised has substantially reduced the training needs of reviewers, who work across different SRs in the same teams, because they work essentially with the very same tools. Furthermore, the fact that the data extraction form goes through all relevant details of the included studies demands that reviewers have a solid understanding of research studies designs. Tasks are facilitated by accessing help hints or external links, present along the forms.

It is unavoidable to compare our approach with the Cochrane‐recommended tools. covidence [10], which has some payment requirements [49], allows importing references, screening and data extraction tasks (with Extraction 1 and Extraction 2), pre‐coded data items (Extraction 2), comparison of outputs between independent reviewers and data export. In some sections, it uses also LLM to extract data items where there are reliable suggestions and the interface of covidence is nice to look at, easy to navigate and covers the functionalities reviewers would require. The main commonality between OSSYRIS and covidence is that both cover importing references, screening, and data extraction with the same sections, equivalent to PICO, plus a section to report findings. covidence, though, does not seem to be integrated with a reference manager or with an analytical and reporting software. The control of data in OSSYRIS is more structured, with pre‐coded questions, validation rules, and calculation fields that streamline data entry, automate decisions, and increase consistency. An important differential aspect is that OSSYRIS forms do not use free text as labels for data items, because this limits standardisation and the possibility to reuse data structures; in this way, post‐data collection coding is not required. Furthermore, our data extraction incorporates the most recent version of the Cochrane ROB tool.

In contrast, EPPI‐Reviewer [11] has a very high level of codification of information and contains multiple data extraction templates. A drawback could be the need to establish strong validation rules, filtering, and internal checks to ensure consistency within and across codes and SRs. We have, though, preferred to develop a single template that eventually allows to interexchange a variety of lists of codes, if needed. OSSYRIS can also be linked to RevMan [7] for analysis and reporting, adapting its data outputs in compatible csv files.

The main limitations of OSSYRIS are: (i) it requires coding skills in XLSForms and in R, although these are common skills in research teams and useful for other types of research activities; (ii) some tasks still require manual operations, although new versions of code and APIs may soon reduce those; (iii) the aesthetical features are limited to those of the XLSForm implementation tool, which is beyond the scope of XLSForms users; (iv) it has no AI functionalities yet, although it provides a robust data scheme as a basis for future AI integration; and (v) as any software, it may contain bugs that we have not detected, yet.

We are currently addressing some of those limitations. We have adapted OSSYRIS to eight SRs (including two overviews of SRs) in less than one day each, including piloting, and we plan to continue using it. We are already incorporating AI components to process the highly structured data extraction dataset, using a purpose‐built tool powered by the Anthropic API (Claude Sonnet 4.6, model string: claude‐sonnet‐4‐6), with the complete dataset (~ 88,800 tokens) embedded in full within each query to minimize hallucination risk through grounded retrieval rather than parametric recall. It is worth noting that OSSYRIS is built on commonly used tools: KoboToolbox is being used by more than 32,000 organisations globally [50] and R is the seventh most popular programming language with millions of users [51].

We expect that OSSYRIS will bring together a community of practice in Cochrane and beyond to add modules (e.g., CERQual [52], TYDieR [53]), enhance functionalities (e.g., online effects calculations) and incorporate AI, to the extent that it fulfils operational challenges in completing SR tasks. Our data extraction form could also be used as a data scheme that journals may request authors to complete, as part of their reporting standards, optimising SR tasks in the future.

## 5 Conclusions

OSSYRIS provides an open source, integrated system to carry out systematic reviews, based on Zotero, KoboToolbox and R. The screening and data extraction forms are fully structured and highly standardised, requiring minimal adaptations to work across different SRs.

We hope that our work may support the promotion of open source and free tools to conduct SR and bring together a community of practice to improve it, in Cochrane and beyond.

## Author Contributions

**Xavier Bosch‐Capblanch:** conceptualization, methodology, software, data curation, investigation, formal analysis, supervision, project administration, writing – original draft, writing – review and editing, funding acquisition. **Christian Auer:** methodology, writing – review and editing. **A. S. M. Sayem:** methodology, writing – review and editing. **Guillaume Deschamps:** methodology, writing – review and editing. **Salvador Camacho:** methodology, writing – review and editing. **Luís Segura:** methodology, writing – review and editing. **Salem Al‐Aidroos:** methodology, writing – review and editing. **George Tsey Sabblah:** methodology, writing – review and editing. **Kaspar Wyss:** writing – review and editing, project administration, supervision, funding acquisition, methodology.

## Conflicts of Interest

The authors declare no conflicts of interest.

## Supporting information

Supporting File 1

Supporting File 2

Supporting File 3

Supporting File 4

## Data Availability

The data that support the findings of this study are openly available in Zenodo at https://zenodo.org/records/20260675.
