# Supplementary material for: An Open‐Source Systematic Reviews Integrated System (OSSYRIS) – Streamlining Processes and Standardising Data Structures
Source: Cochrane Evid Synth Methods. 2026 Jun 5;4(4):e70088. doi: 10.1002/cesm.70088 (PMC13248896; doi:10.1002/cesm.70088)
Supplement: Supplementary file 1 — Supporting File 1 [file CESM-4-e70088-s001.pdf]

		Object	Tasks	Platform	Output	Comments
I		**REQUIREMENTS AND SETUP**				
	(A)	**Cloud**				
	1	Forms, screening and data extraction	Open free account	KoboToolbox	-	Free up to 5,000 submissions a month and 1 GB (https://www.kobotoolbox.org/pricing/); more than enough for most of the reviews.
	2	References	Open free account	Zotero	-	Free up to 300 MB (https://www.zotero.org/storage?id=storage); more than enough if (i) full texts are not uploaded to Zotero server; or (ii) references are downloaded and removed from Zotero after each SR is completed.
	3	Folders for XLSForms files	Create and name folder	SWITCHdrive / OSYRIS / FullText	-	Folder to store full text of studies to avoid using the Zotero server. Can be in any other cloud, to allow collaborative work.
	(B)	**Local machine software**				
	1	Computer	-	Windows	-	Other operating systems should work as well. No specific computational requirement are needed.
	2	Web browser	Install in local machine	Chrome	-	OSYRIS has not been tested in other web browsers.
	3	Spreadsheet software	Install in local machine	MS Excel	-	Other free software is available.
	4	Data software	Install in local machine	R; RStudio	-	Includes libraries for specific functions and APIs.
	5	Reference manager software	Install in local machine	Zotero	-	Optional.
	6	API for reference manager	Download and install API library	R - API Zotero	-	
	7	API for XLSForms	Download and install API library	R - API KoboToolbox	-	
	8	API for reporting	Download and install API library	R - API Office	-	
	9	Writing software	Install in local machine	MS Word	-	Other free software is available. Other outputs include PDF and HTML.
	(C)	**Files**				
	1	XLSForm….xlsx	Obtain from OSYRIS	KoboToolbox	**XLSForm**	xlsx file containing the XLSForm specifications for this specific SR, which is uploaded and deployed in KoboToolbox.
	2	ROB….txt	Upload	GitHub	ROB txt files	Help files that are accessed when filling the XLSForm. Any other platform can be used provided that the appropriate links are used in the XLSForm.
	3	References....txt, .RIS	Obtain from information scientist	R	References txt, RIS files	Produced in R with the appropriate format to be read within the XLSForm using 'pulldata'.
	4	Management....csv	Obtain from OSYRIS	KoboToolbox	Management csv file	Parameters of users; assignments to teams for independently carrying out tasks.
	5	Countries....csv	Obtain from OSYRIS	KoboToolbox	Countries csv file	List of countries with appropriate format to be read within the XLSForm using 'pulldata'.
	6	Logo….png	Produce	KoboToolbox	Logo png file	To be called within the XLSForm in the 'medial' column of 'survey'.
II.		**PREPARATIONS**				
	(A)	**References**				
	1	Reference manager software	Open	Operating system	Zotero opened	
	2	Zotero opened	File > New Library > New Group…	Zotero	Zotero (web) log in	
	3	Zotero (web) log in	Login to Zotero	Zotero (web)	Zotero (web) logged in	
	4	Group in preparation	Choose a name for your group [write the name of the project]	Zotero (web)	Group named	
	5	Group in preparation	Choose Public, Close Membership [check]	Zotero (web)	Group parameters	
	6	Group in preparation	Create Group	Zotero (web)	Group created	
	7	Group in preparation	Library Reading > Any group member [check]	Zotero (web)	Group membership	
	8	Group in preparation	Library Editing > Only group admins [check]	Zotero (web)	Group membership	
	9	Group in preparation	File editing > No group file storage [check]	Zotero (web)	Group parameters	To avoid consuming space in Zotero server (see limitations above).
	10	Group in preparation	Save Settings	Zotero (web)	Group finalised	
	11	Zotero	Close	Zotero (web)	Zotero (web) closed	
	12	Zotero	Sync with zotero.org	Zotero	New group created in Zotero (local)	
	13	Group	Group Libraries > current group [right click] > New Collection… > New Collection > Name: [write name]	Zotero	New collection created	Repeat as many times as needed. Name of sub-collection ending with number of hits (…_##).
	14	References txt, RIS files	File > Import… > A file (BiBTeX, RIS, Zotero RDO, etc.) [check] > Next > File browser > Place imported collections and items into new collection [unchecked];Copy filed to the Zotero storage folder [checked] > Next > Finish	Zotero	Collections by source populated	Repeat as many times as needed.
	15	Collections	Sync with zotero.org	Zotero	Zotero (web) updated	
	16	Zotero collections	Download references	R - API Zotero	Dataframe with references	
	17	Dataframe with references	Codify references	R	References with unique IDN	
	18	Dataframe with references	Cleaning dataframe	R	Dataframe with references cleaned	Create Author-year fields…
	19	Dataframe with references	Update Zotero collections	R - API Zotero	Zotero collections updated	
	20	Collections by source	Copy to a new collection	Zotero	Collections by status	This is not strictly necessary, but keeps the integrity of the collections because deduplication in Zotero operates by merging duplicated references.
	21	References	Run deduplication	Zotero plugin	References unduplicated	Make sure 'Bin' is empty before running duplicates.
	22	References	Populate Status > 1a_AssessRelevant with de-duplicated references	Zotero	Reference ready to process	Manually. Duplicates of references of different 'type' may need to be sorted out manually.
	23	Dataframe with references	Produce references sheet for XLS form	R	References… csv	
	(B)	**XLSForm adaptation**				
	1	**XLSForm**	Adapt form as needed	Local machine	XLSForm adapted	

	Object	Tasks	Platform	Output	Comments
2	XLSForm adapted	Review 'required'	Local machine	XLSForm functional	
3	XLSForm in preparation	Remove 'relevance' in 'calculate' fields.	Local machine	XLSForm functional	Can produce unexpected behaviours.
4	XLSForm in preparation	Check uniqueness of 'names'	Local machine	XLSForm without errors	
5	XLSForm in preparation	Check uniqueness of 'choices'	Local machine	XLSForm without errors	It may not be necessary if repeated codes are allowed in sheet 'settings'.
6	XLSForm in preparation	Check #REF in values	Local machine	XLSForm without errors	
7	XLSForm in preparation	CHK title row in 'settings'	Local machine	XLSForm without errors	
8	XLSForm in preparation	Spell check	Local machine	XLSForm without errors	
9	XLSForm in preparation	ODK XML verification	https://getodk.org/xlsform /	**XLSForm ready to use**	It may happen that not all errors are picked up and then are seen when deploying or loading the form.
(C)	**Launch XLSForms**				
1	-	Login	KoboToolbox	KoboToolbox accessible	
2	-	NEW > Build from scratch > Project name	KoboToolbox	Project named	
3	Project in preparation	… > Sector; Country	KoboToolbox	Countries assigned	
4	Project in preparation	… Create project > Save > Close	KoboToolbox	Project created in 'Draft'	
5	**XLSForm**	Draft > [current project] > Replace form > Upload an XLSForm > Drag and drop the XLSForm file here or click to browser	KoboToolbox	XLSForm uploaded	
6	Project in preparation	Settings > Media > Drag… or click here to browse	KoboToolbox	Media file with references; management file; any images	
7	XLSForm uploaded	Edit in the server	KoboToolbox	XLSForm verified	This is a complementary way of identifying errors; when attempting to editing an error may pop up (e.g. about languages).
8	Project in preparation	Form > Allow submissions to this form without a user name and password	KoboToolbox	Form open for multiple submissions	
9	ROB….txt	Upload	GitHub	ROB help text ready to use	The 'settings' sheet in the XLSForm has to be updated.
10	Management....csv	Settings > Media > Drag… or click here to browse	KoboToolbox	Management parameters ready to be read within XLSForm 'pulldata'	This process will likely be automated using an API in the near future. The 'settings' sheet in the XLSForm has to be updated.
11	Countries....csv	Settings > Media > Drag… or click here to browse	KoboToolbox	Countries codes and names ready to be read within XLSForm 'pulldata'	This process will likely be automated using an API in the near future. The 'settings' sheet in the XLSForm has to be updated.
12	Logo….png	Settings > Media > Drag… or click here to browse	KoboToolbox	Logo ready to be called in column 'media' XLSForm 'survey' sheet	This process will likely be automated using an API in the near future. The 'settings' sheet in the XLSForm has to be updated.
13	References… csv	Settings > Media > Drag… or click here to browse	KoboToolbox	References ready to be read within XLSForm 'pulldata'	This process will likely be automated using an API in the near future. The 'settings' sheet in the XLSForm has to be updated.
14	Project in preparation or updated	Deploy or Redeploy	KoboToolbox	XLSForm available online	
15		*Go to: III·(A)·1 or: III·(B)·1*			
III	**SYSTEMATIC REVIEW TASKS**				
(A)	**Screening**				The same processes apply for 'relevance' and for 'inclusion / exclusion', as references status become classified. Several loops are required to address discrepancies and to allow working in [illegible]
1	**XLSForm**	Fill in the screening form	KoboToolbox	XLSForm data	
2	XLSForm data	Download	R - API KoboToolbox	Screening dataframe	
3	Screening dataframe	Analyse status	R	Dataframe with status	Status: relevant, irrelevant, relevant-unclear.
4	Dataframe with status	Update references dataframe with status	R	References with status	Update SQN (sequential numbering to use in 'pulldata'.
5	References with status	Update Zotero collections	R - API Zotero	Zotero collections updated	
6	References with status	Compare screening A and B	R	List of discrepancies and document	
7	List of discrepancies and document	Address discrepancies	Per SR protocol	References with status	
8	Screening dataframe	Consider 'Comments' in the forms	Manually	Documented ad hoc changes	
9	Screening dataframe	Consider references in the list of references	Manually	Additional references	
10		*Go to: II.·(A)·14*			
11	References with status	Produce PRISMA figure	R	PRISMA figure	The figure will be adapted as work progresses. Automatically produced.
12	References with status	Produce list of included studies	R	Reference list of included studies	As studies become classified as included. Automatically produced.
13	References with status	Produce list of excluded studies	R	Reference list of excluded studies	As studies become classified as excluded. Automatically produced.
14	XLSForm data	Produce table of excluded studies and reasons	R	Table excluded studies	
15	References with status	Produce references csv for KoboToolbox	R	References… csv	
16		*Go to: II.·(C)·13*			
(B)	**Data extraction**				
1	**XLSForm**	Fill in the data extraction form	KoboToolbox	XLSForm data	
2	XLSForm data	Download	R - API KoboToolbox	Data workbook	
3	XLSForm data	Consider 'Comments' in the forms	Manually	Documented ad hoc changes	
4	XLSForm data	Compare data extraction A and B	R	List of discrepancies and document	
5	XLSForm data	Address discrepancies	Manually	Data workbook consistent	
(C)	**Analyses and reporting**				
1	Data workbook consistent	Clean	R	Data workbook clean	
2	Data workbook clean	De-codification	R	Datasets	
3	Datasets	Produce characteristics of included studies	R	Table characteristics of included studies	Automatically produced.
4	Datasets	Produce description of included studies	R	Narrative characteristics of included studies	Automatically produced.
5	Datasets	Produce map of location of studies	R	Map of studies	Automatically produced.
6	Datasets	Analytical outputs	R	Other analytical outputs	Automatically produced.

OSSYRIS University of Basel Swiss TPH

	Object	Tasks	Platform	Output	Comments
7	Datasets and template.docx	Report	R	Report	Automatically and manually produced.

KEY

ROB: Risk of Bias (regardless the specific study design)	
SR: Systematic review	
**XLSForm**	
Files to support XLSForm functionality	
Outputs for SR reporting	Outputs for SR reporting

[END]
