# Supplementary material for: An Open‐Source Systematic Reviews Integrated System (OSSYRIS) – Streamlining Processes and Standardising Data Structures
Source: Cochrane Evid Synth Methods. 2026 Jun 5;4(4):e70088. doi: 10.1002/cesm.70088 (PMC13248896; doi:10.1002/cesm.70088)
Supplement: Supplementary file 2 — Supporting File 2 [file CESM-4-e70088-s002.pdf]

KoboToolbox

CTC | SR 1 Screening

The effects, implementation issues and perceptions of Controlled Temperature Chain (CTC)

OSSYRIS

Swiss TPH

University of Basel

SR_1_Screening [version 20]

Open Source SYstematic Reviews Integrated System *(OSSYRIS)* - DISCLAIMER and LICENSING

OSSYRIS has been developed by the Swiss TPH Team (authors listed below) to support the production of systematic reviews and overviews of systematic reviews. While it has been tested for accuracy and data integrity, the authors cannot guarantee its performance, completeness, or compatibility in all contexts, particularly if modified or used with future versions of XLSForms or relate dplatforms.

The tool is released under a Creative Commons Attribution 4.0 International (CC BY 4.0) license. Users are free to use, adapt, and share the tool, provided appropriate credit is given to the original authors.

Citation: *Bosch-Capblanch X, Deschamps G, Auer C, Sayem A, Camacho S, Segura L, Al-Aidroos S, Sabblah GT, & Wyss K. (2026). Open Source SYstematic Review Integrated System - OSSYRIS (Version 18). Zenodo.* DOI: 10.5281/zenodo.20260675.

OSSYRIS

Welcome! Please, **read everything**, including the 'help' texts that you will find below. All text and elements in this Enketo form have been carefully thought to support the correct filling of the form.

Your code to access the form *	Your name	Your role	Your team
*In this demo version, you can introduce **OSSYRIS-C** or **OSSYRIS-R** as your code to allow you progressing through the form. The value you enter will be remembered only in this device. Ask the project coordinator if unknown.* ▸ *more details* OSSYRIS-C	Alice Whitehead	Coordinator	A and B

Select to show help on **systematic reviews tasks**

- ◯ Yes
- ◉ No

Select to show help on **this form**

- ◯ Yes
- ◉ No

What task are you carrying out? *

- ◯ Assess the relevance of references (titles and abstracts)
- ◉ Assess inclusion or exclusion of relevant references (full text)

Select status of the references to consider *

- ◉ Relevant
- ◯ Excluded
- ◯ Included discrepancy
- ◯ Included unclear
- ◯ Included

Next

Return to Beginning | Go to End

KoboToolbox

CTC | SR 1 Screening

Select reference by author-year: *	Select a reference by sequence from number **1** onwards (the number will be automatically increased up to **1000**): *
1317_Kolwaite_2016	1

Title: Hepatitis B vaccine stored outside the cold chain setting: a pilot study in rural Lao PDR.

Abstract: BACKGROUND: Hepatitis B vaccine birth dose (HepB-BD) was introduced in Lao People's Democratic Republic (Lao-PDR) to prevent perinatal hepatitis B virus transmission. HepB-BD, which is labeled for storage between 2 and 8degreeC, is not available at all health facilities, because of some lack of functional cold chain; however, previous studies show that HepB-BD is stable if stored outside the cold chain (OCC). A pilot study was conducted in Lao-PDR to evaluate impact of OCC policy on HepB-BD coverage., METHODS: During the six month pilot, HepB-BD was stored OCC for up to 28 days in two intervention districts and stored in cold chain in two comparison districts. In the intervention districts, healthcare workers were educated about HepB-BD and OCC storage. A post-pilot survey compared HepB-BD coverage among children born during the pilot (aged 2-8 months) and children born 1 year before (aged 14-20 months)., FINDINGS: In the intervention districts, 388 children aged 2-8 months and 371 children aged 14-20 months were enrolled in the survey; in the comparison districts, 190 children aged 2-8 months and 184 children aged 14-20 months were enrolled. Compared with the pre-pilot cohort, a 27% median increase in HepB-BD (interquartile range [IQR] 58%, p<0.0001) occurred in the pilot cohort in the intervention districts, compared with a 0% median change (IQR 25%, p=0.03) in comparison districts. No adverse reactions were reported., INTERPRETATION: OCC storage improved HepB-BD coverage with no increase in adverse reactions. Findings can guide Lao-PDR on implementation and scale-up options of OCC policy. Copyright Published by Elsevier Ltd.

CRITERIA FOR RELEVANCE

* **Relevant**: focus on **human studies** of **thermostable vaccines** or **CTC**, in real life **programmatic settings** where thermostable vaccines are delivered **outside the cold chain** and/or **CTC** is implemented in the context of non-experimental vaccines.

- *Irrelevant: opinions, editorials, frameworks. Also studies that test thermostability immunogenicity or vaccine efficacy.*

Is this relevant? *

▸ *more details*

(●) Yes ( ) No ( ) I can't tell

At any time you can write here any comment or notes here, so you do not miss anything...

→ Next

Back

Return to Beginning | Go to End

OSSYRIS
