# Supplementary material for: An Open‐Source Systematic Reviews Integrated System (OSSYRIS) – Streamlining Processes and Standardising Data Structures
Source: Cochrane Evid Synth Methods. 2026 Jun 5;4(4):e70088. doi: 10.1002/cesm.70088 (PMC13248896; doi:10.1002/cesm.70088)
Supplement: Supplementary file 3 — Supporting File 3 [file CESM-4-e70088-s003.pdf]

KoboToolbox

CTC | SR 1 Screening

The effects, implementation issues and perceptions of Controlled Temperature Chain (CTC)

OSSYRIS

Swiss TPH

University of Basel

SR_1_Screening [version 20]

Open Source SYstematic Reviews Integrated System *(OSSYRIS)* - DISCLAIMER and LICENSING

OSSYRIS has been developed by the Swiss TPH Team (authors listed below) to support the production of systematic reviews and overviews of systematic reviews. While it has been tested for accuracy and data integrity, the authors cannot guarantee its performance, completeness, or compatibility in all contexts, particularly if modified or used with future versions of XLSForms or related platforms.

The tool is released under a Creative Commons Attribution 4.0 International (CC BY 4.0) license. Users are free to use, adapt, and share the tool, provided appropriate credit is given to the original authors.

Citation: *Bosch-Capblanch X, Deschamps G, Auer C, Sayem A, Camacho S, Segura L, Al-Aidroos S, Sabblah GT, & Wyss K. (2026). Open Source SYstematic Review Integrated System - OSSYRIS (Version 18). Zenodo.* DOI: 10.5281/zenodo.20260675.

Welcome! Please, **read everything**, including the 'help' texts that you will find below. All text and elements in this Enketo form have been carefully thought to support the correct filling of the form.

Your code to access the form *	Your name	Your role	Your team
*In this demo version, you can introduce **OSSYRIS-C** or **OSSYRIS-R** as your code to allow you progressing through the form. The value you enter will be remembered only in this device. Ask the project coordinator if unknown.* ▸ *more details* OSSYRIS-C	Alice Whitehead	Coordinator	A and B

Select to show help on **systematic reviews tasks**

- ○ Yes
- ◉ No

Select to show help on **this form**

- ○ Yes
- ◉ No

What task are you carrying out? *

- ○ Assess the relevance of references (titles and abstracts)
- ◉ Assess inclusion or exclusion of relevant references (full text)

Select status of the references to consider *

- ◉ Relevant
- ○ Excluded
- ○ Included discrepancy
- ○ Included unclear
- ○ Included

Next

Return to Beginning

Go to End

OSSYRIS

KoboToolbox

CTC | SR 1 Screening

Select reference by author-year: *	Select a reference by sequence from number **1** onwards (the number will be automatically increased up to **100**): *
2793_Juan-Giner_2014	2

Title: A cluster randomized non-inferiority field trial on the immunogenicity and safety of tetanus toxoid vaccine kept in controlled temperature chain compared to cold chain

Abstract: BACKGROUND: In resource-poor settings, cold chain requirements present barriers for vaccine delivery. We evaluated the immunogenicity and safety of tetanus toxoid (TT) vaccine in 'Controlled Temperature Chain' (CTC; up to 40 °C for <30 days before administration), compared to standard cold chain (SCC; 2-8 °C). Prior to the study, stability parameters of TT-CTC were shown to meet international requirements. METHODS: A cluster randomized, non-inferiority trial was conducted in Moïssala district, Chad, December 2012-March 2013. Thirty-four included clusters were randomized to CTC or SCC. Women aged 14-49 years, eligible for TT vaccination and with a history of ≤1 TT dose, received two TT doses 4 weeks apart. Participants were blinded to allocation strategy. Tetanus antibody titers were measured using standard ELISA at inclusion and 4 weeks post-TT2. Primary outcome measures were post-vaccination seroconversion and fold-increase in geometric mean concentrations (GMC). Non-inferiority was by seroconversion difference (TTSCC-TTCTC) <5% and ratio of GMCs (TTSCC/TTCTC) <1.5. Adverse events were monitored at health centers and at next contact with participants. RESULTS: A total of 2128 women (CTC=1068; SCC=1060) were recruited. Primary intention to vaccinate analysis included 1830 participants; 272 of these were included in the seroconversion analysis. Seroconversion was reached by >95% of participants; upper 95%CI of the difference was 5.6%. Increases in GMC were over 4-fold; upper 95%CI of GMC ratio was 1.36 in the adjusted analysis. Few adverse events were recorded. CONCLUSIONS: This study demonstrates the immunogenicity and safety of TT in CTC at <40 °C for <30 days. The high proportion of participants protected at baseline results in a reduction of power to detect a 5% non-inferiority margin. However, results at a 10% non-inferiority margin, the comparable GMC increases and vaccine's stability demonstrated in the preliminary phase indicate that CTC can be an alternative strategy for TT delivery in situations where cold chain cannot be maintained.

Is there the full text available in this folder? (**you will need the full text**; look the filename starting with: **2793_Juan-Giner_2014**_shortdescriptors... *

(●) Yes ( ) No

INCLUSION CRITERIA

- **Setting**: **usual delivery of vaccinations** with any strategy (e.g. routine, SIA, PIRI, campaigns...); NOT in experimental studies where vaccination is organised in order to test a particular intervention.
- **Study design**: **experimental** or **quasi-experimental** studies, **controlled before-and-after** study or **interrupted time series**.
- **Participants**: any human being eligible to receive vaccinations; but also people involved in vaccination activities or stakeholders, the perceptions of which may be reported in the study; also health services or system components (e.g. studies that report on supplies).
- **Intervention**: a vaccine has been taken out of the cold-chain a few days before it use for vaccination, commonly (but non only) designated as '**Controlled Temperature Chain**' (CTC). It does NOT include studies that mention (i) that vaccines are taken out of the cold chain in order to be immediately used for vaccination (which is the normal practice); or (ii) that vaccines are taken out of the cold chain for other purposes (e.g. for discarding them).
- **Outcomes**: vaccine status, drop-out, wastage, perceptions, acceptance, resources use (including human resources and supplies), costs...

1) What is the best descriptor of the **contents or topic of the document**? *

(●) Cold chain in the context of routine vaccination
( ) Cold chain in the context of non-routine vaccination
( ) Other uses of cold chain
( ) None of the above

2) What is the best descriptor of the **study setting**? *

(●) Community based care
( ) Primary Health Care
( ) Secondary Care
( ) None of the above

3) What is the best descriptor for the **study design**? *

(●) COMPARES the 'COLD CHAIN INTERVENTION' (CTC...) with the 'STANDARD COLD CHAIN' in different geographical areas or vaccination sites, concurrently during a given time period.
( ) In certain locations or sites, at some point in time, the 'STANDARD COLD CHAIN' was substituted by the 'COLD CHAIN INTERVENTION (CTC...)'; the outcome(s) of interest has been measured MORE THAN ONCE BEFORE (when the 'standard cold chain' was in place) and MORE THAN ONCE AFTER (when the 'cold chain intervention' was in place), in the same locations or sites.
( ) In certain locations or sites, at some point in time, the 'STANDARD COLD CHAIN' was substituted by the 'COLD CHAIN INTERVENTION (CTC...)'; the outcome(s) of interest has been measured ONLY ONCE BEFORE (when the 'standard cold chain' was in place) and ONLY ONCE AFTER (when the 'cold chain intervention' was in place), in the same locations or sites.
( ) The study used QUANTITATIVE METHODS but did NOT COMPARE the 'cold chain intervention' (CTC...) with the 'standard cold chain'.
( ) The study exclusively uses NON-QUANTITATIVE methods.

OSSYRIS

( ) This is a REVIEW OF THE LITERATURE
( ) I can't tell

4) What is the best descriptor for the **main participants** related to the intervention? *

(•) Humans receiving vaccinations or caregivers
( ) Health workers, managers or other staff
( ) Community members
( ) Health services, system components...
( ) Animals
( ) I can't tell

5) What is the best descriptor for the **intervention** or **exposure** reported in the study? *

(•) Vaccines used for vaccination after being outside the cold chain INTENTIONALLY
( ) Vaccines used for vaccination after being outside the cold chain UNINTENTIONALLY
( ) Vaccines used for vaccination after being outside the cold chain JUST SOME MOMENTS BEFORE the vaccination session
( ) Vaccines NEVER put OUTSIDE THE COLD CHAIN
( ) I can't tell

6) What is the best descriptor for the **main outcome(s)** (or results) reported in the study? *

( ) Issues related to vaccine introduction
( ) Issues related to vaccine scale-up
( ) Routine vaccine programme management performance
(•) Only vaccine pharmacological features, efficacy, effectiveness, coverage, wastage... unrelated to vaccine introduction or scale-up.
( ) None of the above
( ) I can't tell

Based on the criteria above, the study **2793_Juan-Giner_2014** is **excluded**. Do you agree with this decision?

(•) Yes
( ) No

Next

Back

Return to Beginning | Go to End

OSSYRIS

KoboToolbox

CTC | SR 1 Screening

If this document (**2793_Juan-Giner_2014**) seems to be a duplicate (i.e. the same document may be retrieved more than once) or closely related to anther study (i.e. sometimes a study may be reported in more than one document), give some explanations here, even if not sure:

Is this document (**2793_Juan-Giner_2014**) an interesting reference to keep an eye into, for any other reason? *

(•) Yes ( ) No

At any time you can write any comment or notes here, so you do not miss anything...

Submit and the next reference will appear.
Thank you very much for your time and collaboration.

Next

Back

Return to Beginning | Go to End

OSSYRIS
