# Supplementary material for: An Open‐Source Systematic Reviews Integrated System (OSSYRIS) – Streamlining Processes and Standardising Data Structures
Source: Cochrane Evid Synth Methods. 2026 Jun 5;4(4):e70088. doi: 10.1002/cesm.70088 (PMC13248896; doi:10.1002/cesm.70088)
Supplement: Supplementary file 4 — Supporting File 4 [file CESM-4-e70088-s004.pdf]

KoboToolbox

CTC | SR 2 Data extraction

The effects, implementation issues and perceptions of Controlled Temperature Chain (CTC)

OSSYRIS

Swiss TPH   University of Basel

SR_2_Data_extraction [version 22]

Open Source SYstematic Reviews Integrated System *(OSSYRIS)* - DISCLAIMER and LICENSING

OSSYRIS has been developed by the Swiss TPH Team (authors listed below) to support the production of systematic reviews and overviews of systematic reviews. While it has been tested for accuracy and data integrity, the authors cannot guarantee its performance, completeness, or compatibility in all contexts, particularly if modified or used with future versions of XLSForms or related platforms.

The tool is released under a Creative Commons Attribution 4.0 International (CC BY 4.0) license. Users are free to use, adapt, and share the tool, provided appropriate credit is given to the original authors.

Citation: *Bosch-Capblanch X, Deschamps G, Auer C, Sayem A, Camacho S, Segura L, Al-Aidroos S, Sabblah GT, & Wyss K. (2026). Open Source SYstematic Review Integrated System - OSSYRIS (Version 18). Zenodo.* DOI: 10.5281/zenodo.20260675.

Welcome! Please, **read everything**, including the 'help' texts that you will find below. All text and elements in this Enketo form have been carefully thought to support the correct filling of the form.

Your code to access the form *	Your name	Your role	Your team
*In this demo version, you can introduce **OSSYRIS-C** or **OSSYRIS-R** as your code to allow you progressing through the form. The value you enter will be remembered only in this device. Ask the project coordinator if unknown.* OSSYRIS-R	Berhane Forest	Reviewer	A

HELP CENTRE - select to read specific help items.

Some text will appear in a box below.

▸ *more details*

- ○ This form
- ○ Risk of Bias
- ○ Known issues and limitations
- ○ Data extraction

Next

Return to Beginning   Go to End

KoboToolbox

CTC | SR 2 Data extraction

1b - Study characteristics | 1983_Wang_L._2007

» (A) Generic characteristics

Language of the main text of the document *

This is not the language of the abstract, which can be in English despite the document being in another language, but that of the full text.

▸ *more details*

English

Is this (or will this be) part of a series of forms to report study **1983_Wang_L._2007**? *

Usually, one document reports one study; however, it could be that one document reports more than one study; for example: similar studies in different countries reported in the same document or a study that has a mix of quantitative and qualitative methods (and both are relevant to the review). In the latter case, you will need to use one form for each study, even if the information is reported in the same document.

▸ *more details*

No

Country *

▸ *more details*

China

What is/are the term(s) that best describe the **setting of the study**? *

▸ *more details*

3 selected

Write a concise label for the **setting of the study**, as designated in the document *

Do not use abbreviations, unconventional characters or redundant terms (e.g. better 'Primary Health Care' than 'Primary Health Care health facilities').

Several health system tiers

Copy and paste (or summarise) the description of the **study setting**.

Leave blank if nothing to report.

Hunan Province between. Three counties within the province: Sangzhl, Longshan and Fenghuang. HBV vaccination is recommended for all children from birth. Within the three counties. all townships situated at least 20 km from the county capital (81 of 116 townships in the county) were chosen to participate in the study. The participating townships Included 957 villages, with total population of about 800,000.

[JBI-XS-7a] Was the study setting described in detail? *

Yes

The start date of a study is important to establish later on issues such as the duration of effects. A study starts when the first participant received an exposure or intervention although this may not be reported as such in the study; instead, other events may be dated and reported. In the drop-down box below, there is a list of events describing the 'start' of the study. Is there any date or time point describing any of the following events (consider the options below *from top to bottom*). *

▸ *more details*

Not specified

What information is available on the **date** of **Not specified**? *

▸ *more details*

at a specific date (only mm/yyyy)

set date *

Click on year to see list of years.

2003-09

The earliest date documenting the start of the study refers to **Not specified** and it is **Sep 2003.**

The end date of a study is important to establish later on issues such as the duration of effects. A study ends when an outcome is measured for the last time, although this may not be reported as such in the study; instead, other events may be dated and reported. In the drop-down box below, there is a list of events describing the 'end' of the study. Is there any date or time point describing any of the following events (consider the options below *from top to bottom*). *

▸ *more details*

Not specified

What information is available that best describes the end of the study? *

▸ *more details*

set date *

Click on year to see list of years.

[illegible]

at a specific date (only mm/yyyy)

The latest date documenting the end of the study refers to **Not specified** and it is **Sep 2004.**

» (B) Study design

Which of the following statements describes best the features of the **study design** in the following questions?

...the number of groups of participants targeted in this study? *

▸ *more details*

- ( ) There is a SINGLE GROUP OF PARTICIPANTS, all of them exposed to the same exposure or intervention (or not exposed or no intervention at all), all along the duration of the study (e.g. survey); note that a study can report several types of participants, but without any comparison between them
- ( ) There is a SINGLE GROUP OF PARTICIPANTS that at some point in time receives an exposure or intervention, the effects of which are COMPARED BEFORE AND AFTER receiving it (e.g. Before-and-After study)
- (•) Participants are split into TWO OR MORE GROUPS according to the DIFFERENT EXPOSURES or INTERVENTIONS they receive, and exposures or interventions are determined by the researchers (e.g. Randomised Controlled Trial)
- ( ) Participants are split into TWO OR MORE GROUPS according to the DIFFERENT EXPOSURES or INTERVENTIONS they receive but researchers do not determine how or when exposures or interventions take place (e.g. Controlled Before-and-After)
- ( ) Participants are split into TWO OR MORE GROUPS according to DIFFERENT CONDITIONS (e.g. having or not having a disease), to ascertain differences in causes or risk factors between groups (e.g. Case-Control study)

...in which sequence were participants exposed to the exposure or intervention? *

▸ *more details*

- (•) All participants start by not experiencing the exposure or intervention and then they are allocated to intervention (or exposure) at the time when they are enrolled (e.g. parallel groups trial)
- ( ) All participants start by not experiencing the exposure or intervention and then they progressively experience it along certain time periods in a way that at the end of the study the same participants have experienced the exposure or the intervention and the comparator (e.g. stepped-wedge trial)
- ( ) Some participants start by not experiencing (and then they are, later on) and some other participants start by experiencing (and then they are not, later on), in a way that at the end of the study all participants have been in the exposure or intervention group and in the comparator group (e.g. cross-over trial)

...the timing when participants in the group were enrolled? *

▸ *more details*

- (•) Participants split into groups and have been enrolled at the same time
- ( ) Participants in one of the groups have experienced the events of interest in the past (i.e. historical controls)

...when was data collected in relation to the planning of the study or protocol? *

▸ *more details*

- (•) The data has been collected prospectively to carry out this research
- ( ) This research is based on existing data (e.g. retrospective)
- ( ) I can't tell

Check *that this information is correct; otherwise, please, write an explanation below.* If there is more than one eligible study design reported in the same document, consider it as a multiple or mixed methods study and use one form for each study design, if eligible.

This study is best described as a **Parallel groups Controlled trial (likely prospective)**.

OSYRIS

You stated that the study compares groups in terms of interventions or exposures. Are you extracting data in terms of 'assignment' (i.e. 'intention to treat' analyses, according to the intervention or exposure group participants where initially assigned) or 'adherence' (i.e. 'per protocol' analysis according the intervention of exposure participants had). *

Check (i) in the protocol and (ii) in the available data.

▸ *more details*

I can't tell ▾

ROBx Is there a protocol defining the main parameters of the study? *

Consider descriptions of participants, interventions, exposures, outcomes, analytical approach, reporting standards...

- ( ) Protocol registered
- ( ) Protocol, not registered, complete
- (•) Protocol, not registered, incomplete
- ( ) Mentioned that no protocol was used
- ( ) No information

» (C) ROB 2 - Domain 1 - randomisation process

You may need to fill in the section 2 - Participants | 1983_Wang_L._2007 to complete this ROB section.

[ROB2 - 1a.1] Was the allocation sequence random? *

Click here if you need help (will open a new window).

▸ *more details*

Probably yes ▾

[ROB2 - 1a.2] Was the allocation sequence concealed until participants were enrolled and assigned to interventions? *

Click here if you need help (will open a new window).

▸ *more details*

No information ▾

[ROB2 - 1a.3] Did baseline differences between intervention groups suggest a problem with the randomization process? *

Click here if you need help (will open a new window).

▸ *more details*

Probably no ▾

Copy and paste (or summarise) the narrative that supports your judgements. *

Use ' instead of " as quotation marks; if PDF: manually remove line breaks.

Rural townships (those lying more than 20 km from the county capital) in the participating counties were randomly divided into three groups ...

The risk of bias in domain '**randomisation process**' is: '**Some concerns**'

If you do not agree with this assessment, state you assessment below and the reasons for disagreement.

Next

Back

Return to Beginning | Go to End

OSSYRIS

KoboToolbox

CTC | SR 2 Data extraction

2 - Participants | 1983_Wang_L._2007

Select to read help text on the role of *participants* in systematic reviews or what the *protocol* of the systematic review says on participants or about the concept of *clusters*.
Some text will appear in a box below.
▸ *more details*

◯ Participants in systematic reviews ◯ Help on clusters ◯ Participants in the protocol

How many different types of **participants** (including **cluster** levels, if any) are enrolled in this study? *Note that participants can be non-human entities (e.g. health facilities, services, health systems components...)* *
Note that this is NOT the number of participants enrolled, but the different TYPES of participants, usually there is one type (e.g. 'mothers were enrolled', would be 1; 'mothers and their partners were enrolled' would be 2). If several strata (e.g. patients by age groups), consider each stratum as a different type of participant and describe all features in this section for each one.

3

How many **cluster nested levels** have been used to enrol participants? *
1 if no clusters at all.

3

» 3.A.1 - Participants: Rural township

1

Write a concise label for the **participants number 1**, as designated in the document. *
Do not use abbreviations, unconventional characters or redundant terms (e.g. better 'Adolescents school' than 'Adolescents enrolled in schools').

Rural township

What is the best descriptor for participants **Rural township**? *
▸ *more details*

Geographical or administrative area ▾

Copy and paste (or summarise) the description of **participants Rural township** as it appears in the document.
Leave blank if nothing to report.

Those lying more than 20 km from the county capital

Copy and paste (or summarise) any other inclusion or exclusion criteria for **participants Rural township**.
Leave blank if nothing to report.

Those lying more than 20 km from the county capital

What is the cluster level for participants **Rural township**? *
1: top or unique level, 2: second nested level...

1

Is participant type **Rural township** used to select or enrol other participants? *
Examples: 12 health facilities (this level) used to select patients within them (answer 'Yes'); 4 districts (this level) where individuals are selected from, regardless their district of residence (here districts are just 'study areas'; typically data is not aggregated by district) (answer 'No').
▸ *more details*

◉ Yes ◯ No

How many **Rural township** have been enrolled or selected? *
*These are the **total**, across all clusters (if applicable) or across all groups (if applicable). Note that this is not necessarily the number that appears in the 'Results' since some participants may have been dropped. Write -7 if missing.*

-7

How were participants of type **Rural township** enrolled? *
▸ *more details*

I can't tell ▾

Will you collect data on outcomes specifically for participant **Rural township**? *
Note that there may be participants receiving the intervention (e.g. pregnant women) but outcomes may be assessed in other participants (e.g. birth weight in babies).
▸ *more details*

◯ Yes ◉ No

Do you think that participants of the type Rural township are **representative** of the wider group of participants of the same type that could be found in this or similar settings? *
▸ *more details*

Yes ▾

» 3.A.2 - Participants: Village

2

Write a concise label for the **participants number 2**, as designated in the document. *
Do not use abbreviations, unconventional characters or redundant terms (e.g. better 'Adolescents school' than 'Adolescents enrolled in schools').

Village

What is the best descriptor for participants **Village**? *
▸ *more details*

Geographical or administrative area ▾

Copy and paste (or summarise) the description of **participants Village** as it appears in the document.
Leave blank if nothing to report.

No details.

Copy and paste (or summarise) any other inclusion or exclusion criteria for **participants Village**.
Leave blank if nothing to report.

No details.

What is the cluster level for participants **Village**? *
1: top or unique level, 2: second nested level...

2

OSSYRIS

Examples: 12 health facilities (this level) used to select patients within them (answer 'Yes'); 4 districts (this level) where individuals are selected from, regardless their district of residence (here districts are just 'study areas'; typically data is not aggregated by district) (answer 'No').

▸ more details

(●) Yes ( ) No

selected?

*These are the **total**, across all clusters (if applicable) or across all groups (if applicable). Note that this is not necessarily the number that appears in the 'Results' since some participants may have been dropped. Write -7 if missing.*

120

How were participants of type **Village** enrolled? *

▸ more details

Random, probability proportional to s…

Copy and paste (or summarise) the description of how **participants Village** were enrolled.

Leave blank if nothing to report.

No details.

Will you collect data on outcomes specifically for participant **Village**? *

Note that there may be participants receiving the intervention (e.g. pregnant women) but outcomes may be assessed in other participants (e.g. birth weight in babies).

▸ more details

( ) Yes (●) No

Do you think that participants of the type Village are **representative** of the wider group of participants of the same type that could be found in this or similar settings? *

▸ more details

Yes

3

» 3.A.3 - Participants: Infants - children

Write a concise label for the **participants number 3**, as designated in the document. *

Do not use abbreviations, unconventional characters or redundant terms (e.g. better 'Adolescents school' than 'Adolescents enrolled in schools').

Infants - children

What is the best descriptor for participants **Infants - children**? *

▸ more details

Community member

Copy and paste (or summarise) the description of **participants Infants - children** as it appears in the document.

Leave blank if nothing to report.

No details.

Copy and paste (or summarise) any other inclusion or exclusion criteria for **participants Infants - children**.

Leave blank if nothing to report.

No details.

What is the cluster level for participants **Infants - children**? *

1: top or unique level, 2: second nested level...

3

How many **Infants - children** have been enrolled or selected? *

*These are the **total**, across all clusters (if applicable) or across all groups (if applicable). Note that this is not necessarily the number that appears in the 'Results' since some participants may have been dropped. Write -7 if missing.*

6988

How were participants of type **Infants - children** enrolled? *

▸ more details

I can't tell

Will you collect data on outcomes specifically for participant **Infants - children**? *

Note that there may be participants receiving the intervention (e.g. pregnant women) but outcomes may be assessed in other participants (e.g. birth weight in babies).

▸ more details

(●) Yes ( ) No

What was the age criteria to select or enrol **participants Infants - children**? *

▸ more details

2 selected

minimum age *

Write -7 if no minimum age.

0

units *

▸ more details

days

maximum age *

Write -7 if no minimum age.

20

units *

▸ more details

months

[PROGRESS-Plus] Which of the following features **are explicitly mentioned** in the text as being considered in the **enrolment** or **selection** of participants of the type **Infants - children**? *

▸ more details

- [x] Place or residence
- [x] Race, ethnicity, culture, language
- [ ] Occupation
- [ ] Gender, sex
- [ ] Religion
- [ ] Education
- [x] Socio-economic status
- [ ] Social capital
- [ ] None of those

Place of residence *

▸ more details

Rural

Race, ethnicity, culture, language *

▸ more details

Asian

Socio-economic status *

▸ more details

Low income / wealth

The features found in the text are: **Rural | Asian | | | | Poor |** (*you can add or remove items by selecting the corresponding feature in the previous lists*)

Do you think that participants of the type Infants - children are **representative** of the wider group of participants of the same type that could be found in this or similar settings? *

▸ more details

Yes

The participants of the type **Infants - children**, as described in the study, are they similar enough to the participants of interest, as defined in the protocol of this review? *

▸ more details

Yes, because they are practically identical

» 2.B ROB2 - Domain 1b - Selection of participants in cluster study designs

OSSYRIS

[ROB2-1b.1] Were all the individual participants identified and recruited (if appropriate) before randomization of clusters? *	[ROB2-1b.2] Is it likely that selection of individual participants was affected by knowledge of the intervention assigned to the cluster? *	[ROB2-1b.3] Were there baseline imbalances that suggest differential identification or recruitment of individual participants between intervention groups? *
Click here if you need help (will open a new window). ▸ *more details*	*Click here if you need help (will open a new window).* ▸ *more details*	*Click here if you need help (will open a new window).* ▸ *more details*
Probably no	Probably no	No

Copy and paste (or summarise) the narrative that supports your judgements. *

Use ['] (simple quotation marks) instead of ["] (double quotation marks), if needed. If pasting from PDF: manually remove line breaks, please.

There Is no Information on how Infants were recruited. It would seem that all Infants born In the selected townships would have been enrolled. Then, the outcomes were measured in a sample of those.

The risk of bias in domain '**selection of participants**' in a cluster design is: '**Some concerns**'

If you do not agree with this assessment, state you assessment below and the reasons for disagreement.

Next

Back

Return to Beginning

Go to End

OSSYRIS

CTC | SR 2 Data extraction

3 - Intervention or exposure | 1983_Wang_L._2007

Select to red help on **'Interventions'** or '**Exposures**'.

Some text will appear in a box below.

▸ *more details*

◯ Interventions or exposures in systematic reviews ◯ Interventions or exposures in the protocol

You indicated that the study compares the effects of exposures or interventions between groups or before and after. How many different exposures or interventions are studied (**without** considering here a '**control**' or '**usual care**' group)? *

Different interventions refer to interventions being assessed or compared and administered separately; a multifaceted intervention (e.g. patient education and cancer screening) is (i) a unique intervention if it is administered together to a group of patients or (ii) it would be 'several' interventions if one group of participants received 'education' only and another group received 'screening' only.

3

Is there a **control** or **usual care** used as comparison? *

This is NOT another intervention or an intervention with different intensity or dosage; it is what is considered as routine or usual care.

▸ *more details*

◯ Yes ◉ No

» 3.A Description of intervention(s) or exposure(s)

1

Write a concise label for the **intervention or exposure number 1**, as designated in the document. *

Do not use abbreviations, unconventional characters or redundant terms (e.g. better 'Measles vaccination' than 'Administration of vaccine against measles').

HepB amp. cold chain Hospital

Copy and paste (or summarise) the description of the **HepB amp. cold chain Hospital**

Leave blank if nothing to report.

HepB vaccine used was provided in ampoules. stored within the cold chain and administered using auto-disable syringes to infants in township hospitals. HepB vaccination practices in these townships differed from pre-study practices only in that there was additional awareness raising about the importance of vaccination. Parents of children born outside the hospital were requested by village doctors to bring their children to the hospital as soon after birth as possible for the first dose of HepB vaccine.

What term(s) best describes **HepB amp. cold chain Hospital** *

▸ *more details*

Standard cold chain

What vaccine(s) is(are) involved? *

▸ *more details*

HBV birth dose

The intervention (or exposure) **HepB amp. cold chain Hospital**, as described in the study, is similar enough to the intervention (or exposure) of interest, as defined in the protocol of the systematic review. *

▸ *more details*

Hardly or not at all

2

Write a concise label for the **intervention or exposure number 2**, as designated in the document. *

Do not use abbreviations, unconventional characters or redundant terms (e.g. better 'Measles vaccination' than 'Administration of vaccine against measles').

HepB amp. outside CC - VHW

Copy and paste (or summarise) the description of the **HepB amp. outside CC - VHW**

Leave blank if nothing to report.

HepB vaccine was provided in ampoules, stored out of the cold chain in villages, and administered using auto-disable syringes by village based health workers to Infants in their homes.

What term(s) best describes **HepB amp. outside CC - VHW** *

▸ *more details*

Controlled Temperature Chain

What vaccine(s) is(are) involved? *

▸ *more details*

HBV birth dose

The intervention (or exposure) **HepB amp. outside CC - VHW**, as described in the study, is similar enough to the intervention (or exposure) of interest, as defined in the protocol of the systematic review. *

▸ *more details*

Yes, because there are differences but do not compromise applicability of evidence

3

Write a concise label for the **intervention or exposure number 3**, as designated in the document. *

Do not use abbreviations, unconventional characters or redundant terms (e.g. better 'Measles vaccination' than 'Administration of vaccine against measles').

HepB-Unlject • CHW

Copy and paste (or summarise) the description of the **HepB-Unlject • CHW**

Leave blank if nothing to report.

HepB vaccine was provided in Uniject devices, stored out of the cold chain In villages and administered by village-based health workers to infants in their homes.

What term(s) best describes **HepB-Unlject • CHW** *

What vaccine(s) is(are) involved? *

Controlled Temperature Chain	HBV birth dose

The intervention (or exposure) **HepB-Unlject • CHW**, as described in the study, is similar enough to the intervention (or exposure) of interest, as defined in the protocol of the systematic review. *

▸ *more details*

Yes, because there are differences but do not compromise applicability of evidence

Describe whether there is/are any other co-intervention(s) or co-exposure(s) that may affect the results in any participants' group?

Leave blank if nothing to report

Interventions differ in the type of health care provider who delivers them. In group 1, It is In the hospital. While in the other two groups, they are CHW.

» 3.B Allocation of participants (numbers)

These are the number of participants that were **actually assigned** to each exposure or intervention or group, as reported in the document. In some studies, this may not be reported as such, but instead the study reports the number of participants that have entered the analyses in the results: in that case, write -7, because we cannot be sure that the numbers of participants in the analyses reflect all those enrolled, since some may abandon or be lost to follow up (note that you will be able to enter the number of participants in the analyses later on, in another section of this form).

Rural township	HepB amp. cold chain Hospital * -7	HepB amp. outside CC - VHW * -7	HepB-Unlject • CHW * -7	Total Unknown	Enrolled -7	1
Village	HepB amp. cold chain Hospital * 40	HepB amp. outside CC - VHW * 40	HepB-Unlject • CHW * 40	Total 120	Enrolled 120	2
Infants - children	HepB amp. cold chain Hospital * -7	HepB amp. outside CC - VHW * -7	HepB-Unlject • CHW * -7	Total Unknown	Enrolled 6988	3

'**Total**' and '**Enrolled**' should be equal; if not, explain why below.

» 3.C ROB 2 - Domain 2 (part 1) - deviations from the intended interventions (assignment or adherence)

[ROB2 - 2.1a] Were participants aware that they were in a trial? *

Click here if you need help (will open a new window).

▸ *more details*

Probably yes

[ROB2 - 2.1b] Were participants aware of their assigned intervention during the trial? *

Click here if you need help (will open a new window).

▸ *more details*

Yes

[ROB2 - 2.2] Were carers and people delivering the interventions aware of participants' assigned intervention during the trial? *

Click here if you need help (will open a new window).

▸ *more details*

Yes

[ROB2 - 2.3 - Adherence] Were important non-protocol interventions balanced across intervention groups? *

Click here if you need help (will open a new window).

▸ *more details*

No information

Copy and paste (or summarise) the narrative that supports your judgements. *

Use ['] (simple quotation marks) instead of ["] (double quotation marks), if needed. If pasting from PDF: manually remove line breaks, please.

There were some awareness activities. The interventions could no be blinded to participants.

➔ Next

Back

COSSYRIS

KoboToolbox

CTC | SR 2 Data extraction

4 - Outcomes | 1983_Wang_L._2007

Select to read help text on the role of *outcomes* in reviews or what the *protocol* of the review says on outcomes.
Some text will appear in a box below.
▸ *more details*

○ Outcomes in systematic reviews ○ Outcomes in the protocol

How many outcomes of interest are reported in the document? *

2

1

» 4·1·A - Description of outcome: HepB timeliness

Write a concise label for the **outcome number outcome number 1**, as designated in the document. *
If the outcome is continuous or ordinal, do NOT use terms for change (e.g. 'increase', 'decrease', 'change'...) | If the outcome is categorical (e.g. vaccinated yes / no, income high / moderate / low) mention the category used to report this outcome (e.g. 'vaccination received', or 'income high') | Do no include details about participants (this is detailed elsewhere).

HepB timeliness

Select what better describes the outcome **HepB timeliness** *
▸ *more details*

Vaccination performance

What type of outcome is **HepB timeliness** *
▸ *more details*

Coverage or utilisation

To which participants does outcome **HepB timeliness** refer to? *
▸ *more details*

☑ Infants - children

How was **HepB timeliness** assessed? *
▸ *more details*

Not stated

Copy and paste (or summarise) the description of the **HepB timeliness** (this is NOT the result of the outcome -which comes later- but the 'meaning' of the outcome).
Leave blank if nothing to report.

HepB given within the first 24 hours after birth.

How important do you think is outcome **HepB timeliness** to eventually make a policy or practical decision? *
▸ *more details*

Critical

What type of values has outcome **HepB timeliness**? *
You can consider as 'qualitative' those outcomes that are composite and that will not be included in further quantitative analyses in the SR (e.g. 'Perceptions of users' could classified as qualitative and be reported in section 5 as a narrative: '15% were satisfied with health workers, 8% did not understand the treatment and 67% were generally satisfied').
▸ *more details*

Categorical or dichotomous (e.g. colours, high / low income, yes/n…

How many categories are defined? *
Write -7 if missing or unknown.

2

Higher values or more of outcome **HepB timeliness**, imply... *
▸ *more details*

More benefits

Is the unit of analysis the same as the participants *
If different from the 'participants'. Consider that some outcomes may be repeatedly measured and reported more than once for the same participant (e.g. a study with 10 participants but individually reports 3 blood pressure measures for each participant, making 10 x 3 the number of observations, despite that there are only 10 participants; in this case the unit of analysis would be 'person-times').
▸ *more details*

◉ Yes ○ No

The outcome, **HepB timeliness** as described in the study and in the timing assessed, is similar enough to an outcome of interest and the timing of its assessment, as defined in the protocol of the review. *
▸ *more details*

Yes, because they are practically identical

4·1·B - ROB2 - Domain 3 - **Missing outcome data | HepB timeliness**

[ROB2 - 3.1] Were data for this outcome available for all, or nearly all, participants randomized? *
Click here if you need help (will open a new window).
▸ *more details*

Probably yes

[ROB2 - 3.1a] Were data for this outcome available for all clusters that recruited participants? *
Click here if you need help (will open a new window).
▸ *more details*

Yes

[ROB2 - 3.1b] Were data for this outcome available for all, or nearly all, participants within clusters? *
Click here if you need help (will open a new window).
▸ *more details*

Probably yes

OSSYRIS

Copy and paste (or summarise) the narrative that supports your judgements.
Use ['] (simple quotation marks) instead of ["] (double quotation marks), if needed. If pasting from PDF: manually remove line breaks, please.

The methods state: 'During the study period, 6988 infants were born in the three study groups. In the baseline coverage survey, there were 1202 infants, with 42.4% born at home. In the follow-up coverage survey, there were also 1202 infants, 38.5% born at home. In the serological survey, there were 606 infants'.
They run a survey in each village. Strictly speaking. there is no data for all participants randomised (3.1), but the study was not designed to assess the outcome in every vaccinated child either.

The risk of bias in domain '**missing outcome data**' (cluster trial) is: '**Low risk**'
If you do not agree with this assessment, state you assessment below and the reasons for disagreement.

4·1·B - ROB 2 - Domain 4 - **Measurement of the outcome | HepB timeliness**

[ROB2 - 4.1] Was the method of measuring the outcome **HepB timeliness** inappropriate?	[ROB2 - 4.2] Could the measurement or ascertainment of the outcome **HepB timeliness** have differed between intervention groups?	[ROB2 - 4.3a] Were outcome **HepB timeliness** assessors aware that a trial was taking place?	[ROB2 - 4.3b] Were the outcome **HepB timeliness** assessors aware of the intervention received by study participants?	[ROB2 - 4.4] Could the assessments of the outcome **HepB timeliness** have been influenced by knowledge of intervention received?	[ROB2 - 4.5] Is it likely that the assessments of the outcome **HepB timeliness** were influenced by knowledge of intervention received?
Click here if you need help (will open a new window). ▸ more details	*Click here if you need help (will open a new window).* ▸ more details	*Click here if you need help (will open a new window).* ▸ more details	*Click here if you need help (will open a new window).* ▸ more details	*Click here if you need help (will open a new window).* ▸ more details	*Click here if you need help (will open a new window).* ▸ more details
Probably no	No	Probably yes	Probably yes	Probably no	No

[ROB2 - 4.5] Is it likely that the assessments of the outcome **HepB timeliness** were influenced by knowledge of intervention received?

They do not explain how they measure the outcome, but cite reference of a methodological paper (reference 14), not a bit outdated.
The Intervention could not be blinded and there are no explanations about whether assessors were blinded or not; however, the fact that the recipients of intervention were neither blind, does not allow us to assume that assessors were.

The risk of bias in domain '**measurement of the outcome**' is: '**Low risk**'
If you do not agree with this assessment, state you assessment below and the reasons for disagreement.

2

» 4·2·A - Description of outcome: AntI-HBs titres after 3 doses

Write a concise label for the **outcome number outcome number 2**, as designated in the document.
If the outcome is continuous or ordinal, do NOT use terms for change (e.g. 'increase', 'decrease', 'change'...) | If the outcome is categorical (e.g. vaccinated yes / no, income high / moderate / low) mention the category used to report this outcome (e.g. 'vaccination received', or 'income high') | Do no include details about participants (this is detailed elsewhere).

AntI-HBs titres after 3 doses

Select what better describes the outcome **AntI-HBs titres after 3 doses**
▸ more details

Vaccination performance

What type of outcome is **AntI-HBs titres after 3 doses**
▸ more details

Coverage or utilisation

To which participants does outcome **AntI-HBs titres after 3 doses** refer to?
▸ more details

☑ Infants - children

How was **AntI-HBs titres after 3 doses** assessed?
▸ more details

Measuring device

Copy and paste (or summarise) the description of the **AntI-HBs titres after 3 doses** (this is NOT the result of the outcome -which comes later- but the 'meaning' of the outcome).
Leave blank if nothing to report.

Specimens were tested by radioimmunoassay (Bei Fang Biological Technical Insti tute, Beijing) at the National Vaccine and Serum Institute (NVSI) for HBsAg, antibody to HBsAg (anti-HBs), and antibody to HBV core antigen (anti-HBc). For samples with undetectable titres, a value of 2.0 mIU/ml (the lower limit of detection of the assay) was assigned when calculating the geometric mean titre. The antI-HBs ti tres were estimated by serial dilution.

How important do you think is outcome **AntI-HBs titres after 3 doses** to eventually make a policy or practical decision?
▸ more details

Important but not critical

What type of values has outcome **AntI-HBs titres after 3 doses**?
You can consider as 'qualitative' those outcomes that are composite and that will not be included in further quantitative analyses in the SR (e.g. 'Perceptions of users' could classified as qualitative and be reported in section 5 as a narrative: '15% were satisfied with health workers, 8% did not understand the treatment and 67% were generally satisfied').
▸ more details

Continuous, dimensional (e.g. any value, including decimals, with units like Kg, mm…

What are the units of measure of **AntI-HBs titres after 3 doses**?
For example: Kg, mmHg... Use standard terms.

mIU/ml

AVOSSYRIS

**Antl-HBs titres after 3 doses** can take? *Write -7 if unknown* 0	*Write -7 if unknown* -7	outcome **Antl-HBs titres after 3 doses**, imply... ▸ *more details* More benefits	as the participants *If different from the 'participants'. Consider that some outcomes may be repeatedly measured and reported more than once for the same participant (e.g. a study with 10 participants but individually reports 3 blood pressure measures for each participant, making 10 x 3 the number of observations, despite that there are only 10 participants; in this case the unit of analysis would be 'person-times').* ▸ *more details* (●) Yes ( ) No

The outcome, **Antl-HBs titres after 3 doses** as described in the study and in the timing assessed, is similar enough to an outcome of interest and the timing of its assessment, as defined in the protocol of the review. *

▸ *more details*

Partially

4·2·B - ROB2 - Domain 3 - **Missing outcome data | Antl-HBs titres after 3 doses**

[ROB2 - 3.1] Were data for this outcome available for all, or nearly all, participants randomized? *	[ROB2 - 3.1a] Were data for this outcome available for all clusters that recruited participants? *	[ROB2 - 3.1b] Were data for this outcome available for all, or nearly all, participants within clusters? *	[ROB2 - 3.2] Is there evidence that the result was not biased by missing outcome data? *
Click here if you need help (will open a new window). ▸ *more details* No	*Click here if you need help (will open a new window).* ▸ *more details* Probably yes	*Click here if you need help (will open a new window).* ▸ *more details* No	*Click here if you need help (will open a new window).* ▸ *more details* No

[ROB2 - 3.3] Could missingness in the outcome depend on its true value? *

Click here if you need help (will open a new window).

▸ *more details*

No

Copy and paste (or summarise) the narrative that supports your judgements. *

Use ['] (simple quotation marks) instead of ["] (double quotation marks), if needed. If pasting from PDF: manually remove line breaks, please.

Is not mentioned how the 606 children included in the serological survey were selected.

The risk of bias in domain '**missing outcome data**' (cluster trial) is: '**Low risk**'

If you do not agree with this assessment, state you assessment below and the reasons for disagreement.

4·2·B - ROB 2 - Domain 4 - **Measurement of the outcome | Antl-HBs titres after 3 doses**

[ROB2 - 4.1] Was the method of measuring the outcome **Antl-HBs titres after 3 doses** inappropriate? *	[ROB2 - 4.2] Could the measurement or ascertainment of the outcome **Antl-HBs titres after 3 doses** have differed between intervention groups? *	[ROB2 - 4.3b] Were the outcome **Antl-HBs titres after 3 doses** assessors aware of the intervention received by study participants? *
Click here if you need help (will open a new window). ▸ *more details* Yes	*Click here if you need help (will open a new window).* ▸ *more details* No	*Click here if you need help (will open a new window).* ▸ *more details* Probably no

[ROB2 - 4.5] Is it likely that the assessments of the outcome **Antl-HBs titres after 3 doses** were influenced by knowledge of intervention received? *

Laboratory tests described above. No further details In the text.

The risk of bias in domain '**measurement of the outcome**' is: '**Low risk**'

If you do not agree with this assessment, state you assessment below and the reasons for disagreement.

» 4.C Timing outcomes were measured

How many times has/have outcome(s) being assessed and you wish to report here, based on the eligibility of timing, according to the review protocol? *

An outcome could have been measured many times, but you may be interested only in the first and last measures; in this example, enter '2' in this field. If different outcomes are measured at different points in time, enter the number of points in time across all outcomes you are interested in (you will be able to select specific timings later on).

2

What information is available on this **date** or **timing**? * 1

▸ *more details*

at baseline

What information is available on this **date** or **timing**? * 2

▸ *more details*

at end-line

SOSSYRIS

OSSYRIS

KoboToolbox

CTC | SR 2 Data extraction

5 - Results | 1983_Wang_L._2007

How many **results** will you be reporting? *

*In this section you can report individual data items or data that describe the effects of an exposure or intervention in the outcomes of interest, either **comparing** different exposures or interventions and/or different times or describing **trends**.*

3

Next

Back

Return to Beginning | Go to End

OSSYRIS

CTC | SR 2 Data extraction

5 - Results | 1983_Wang_L._2007

» 5·1·A - HepB timeliness [in Infants - children]: HepB amp. cold chain Hospital vs HepB amp. outside CC - VHW | several points in time 1

Select the type of **data** that you are reporting here *
▸ *more details*

Differences between two exposures or interventions, at a given point in time

Select the **participants** you want to report about *	Select the **interventions** to compare *	Outcomes *	Timing *
▸ *more details*	▸ *more details*	▸ *more details*	▸ *more details*
(●) Infants - children	[✔] HepB amp. cold chain Hospital<br>[✔] HepB amp. outside CC - VHW<br>[ ] HepB-Unlject • CHW	(●) HepB timeliness<br>( ) AntI-HBs titres after 3 doses (mIU/ml)	( ) at baseline<br>(●) at end-line

1 - Are you reporting data for participants: **Infants - children** on outcome **HepB timeliness** in group **HepB amp. cold chain Hospital** in time ? *
▸ *more details*

(●) Yes ( ) No

What statistic is used to summarise **HepB timeliness**? * 1
Note that if you are reporting an outcome in different ways (e.g. 'birth weight' as a continuous variable in Kg and as a proportion of participants above or below a certain threshold), then these should be considered as two different outcomes.
▸ *more details*

( ) Sum ( ) Count (●) Proportion (%) ( ) Other

N of **Infants - children** * *In sample (write -7 if unknown)*	Proportion (%) *	SD *	SE *	95% CI L *	95% CI U *	Statistical test * *For example: F, t, X2...*	Statistical test value *	p-value *
401	57.9	-7	-7	50.3	65.4	-7	-7	-7

Details on any **adjustment** carried out by authors, consider as well whether there is any **modelling** in the estimates of the effects. *
For example, for clustering.
The text mentions that clustering has been taken into account.

2 - Are you reporting data for participants: **Infants - children** on outcome **HepB timeliness** in group **HepB amp. outside CC - VHW** in time ? *
▸ *more details*

(●) Yes ( ) No

What statistic is used to summarise **HepB timeliness**? * 2
Note that if you are reporting an outcome in different ways (e.g. 'birth weight' as a continuous variable in Kg and as a proportion of participants above or below a certain threshold), then these should be considered as two different outcomes.
▸ *more details*

( ) Sum ( ) Count (●) Proportion (%) ( ) Other

N of **Infants - children** * *In sample (write -7 if unknown)*	Proportion (%) *	SD *	SE *	95% CI L *	95% CI U *	Statistical test * *For example: F, t, X2...*	Statistical test value *	p-value *
401	67.8	-7	-7	61.5	74.2	-7	-7	-7

Details on any **adjustment** carried out by authors, consider as well whether there is any **modelling** in the estimates of the effects. *
For example, for clustering.
The Proportion (%) of HepB timeliness assessed in 401Infants - children belonging to the group undefined, in time at end-line: 67.8 [SI

Are you reporting data for participants: **Infants - children** on *differences* of outcome **HepB timeliness** between **HepB amp. cold chain Hospital** and **HepB amp. outside CC - VHW** in time *
▸ *more details*

(●) Yes ( ) No

What statistic is used to assess the effects in **HepB timeliness**? * 3
Note that if you are reporting an outcome in different ways (e.g. 'birth weight' as a continuous variable in Kg and as a proportion of participants above or below a certain threshold), then these should be considered as two different outcomes.
▸ *more details*

(●) Proportions difference ( ) Absolute risk difference ( ) Hazard Ratio
( ) Risk Ratio ( ) Odds Ratio ( ) Other

MOSSYRIS

N of **Infants - children** *In sample (write -7 if unknown)* *	Proportions difference *	SD *	SE *	95% CI L *	95% CI U *	Statistical test *For example: F, t, X2...* *	Statistical test value *	p-value *
802	-7	-7	-7	-7	-7	X2	-7	<0.05

Details on any **adjustment** carried out by authors, consider as well whether there is any **modelling** in the estimates of the effects. *
For example, for clustering.

The Proportions difference of Antl-HBs titres after 3 doses (mIU/ml) assessed in 802Infants - children belonging to the group undefine

» 5·1·B - ROB 2 - Domain 2 (part 2) - Missing outcome data | HepB timeliness

[ROB2 - 2.6 - Adherence] Was an appropriate analysis used to estimate the effect of adhering to the intervention? *
Click here if you need help (will open a new window).
▸ more details

Yes

Copy and paste (or summarise) the narrative that supports your judgements. *
Use ['] (simple quotation marks) instead of ["] (double quotation marks), if needed. If pasting from PDF: manually remove line breaks, please.

The text reports on cold chain Issues and wastage due to overexposure to heat, suggesting the hospitals and community health workers implemented the intervention as expected.

The risk of bias in domain '**deviations from the intended interventions (adhering to intervention)**' is: '**Some concerns**'
If you do not agree with this assessment, state you assessment below and the reasons for disagreement.

» 5·1·B - ROB 2 - Domain 5 - Selection of the reported result | HepB timeliness

[ROB2 - 5.1] Were the data that produced this result analysed in accordance with a pre-specified analysis plan that was finalized before unblinded outcome data were available for analysis? *
Click here if you need help (will open a new window).
▸ more details

No information

[ROB2 - 5.2] Is the numerical result being assessed likely to have been selected, on the basis of the results, from multiple eligible outcome measurements (e.g. scales, definitions, time points) within the outcome domain? *
Click here if you need help (will open a new window).
▸ more details

Probably no

[ROB2 - 5.3] Is the numerical result being assessed likely to have been selected, on the basis of the results, from multiple eligible analyses of the data? *
Click here if you need help (will open a new window).
▸ more details

Probably no

Copy and paste (or summarise) the narrative that supports your judgements. *
Use ['] (simple quotation marks) instead of ["] (double quotation marks), if needed. If pasting from PDF: manually remove line breaks, please.

The outcome Is well defined and consistent with standards. There is no much room to select results.

The risk of bias in domain '**measurement of the outcome**' is: '**Some concerns**'
If you do not agree with this assessment, state you assessment below and the reasons for disagreement.

» OVERALL RISK OF BIAS for HepB timeliness.

The overall judgment of the risk of bias for this outcome-comparison is **Some concerns**. If you disagree with this assessment, please, write an explanation below, or leave blank otherwise.
Disagreement may come from the fact that the study is judged to have some concerns for multiple domains in a way that substantially lowers confidence in the result.

→ Next

Back

Return to Beginning | Go to End

OSSYRIS

CTC | SR 2 Data extraction

5 - Results | 1983_Wang_L._2007

» 5·2·A - HepB timeliness [in Infants - children]: HepB amp. cold chain Hospital vs HepB-UnIject • CHW | several points in time 2

Select the type of **data** that you are reporting here *
▸ more details

Differences between two exposures or interventions, at a given point in time

Select the **participants** you want to report about *	Select the **interventions** to compare *	Outcomes *	Timing *
▸ more details	▸ more details	▸ more details	▸ more details
(●) Infants - children	[✔] HepB amp. cold chain Hospital<br>[ ] HepB amp. outside CC - VHW<br>[✔] HepB-UnIject • CHW	(●) HepB timeliness<br>( ) AntI-HBs titres after 3 doses (mIU/ml)	( ) at baseline<br>(●) at end-line

1 - Are you reporting data for participants: **Infants - children** on outcome **HepB timeliness** in group **HepB amp. cold chain Hospital** in time ? *
▸ more details
(●) Yes ( ) No

What statistic is used to summarise **HepB timeliness**? * 1
Note that if you are reporting an outcome in different ways (e.g. 'birth weight' as a continuous variable in Kg and as a proportion of participants above or below a certain threshold), then these should be considered as two different outcomes.
▸ more details
( ) Sum ( ) Count (●) Proportion (%) ( ) Other

N of **Infants - children** * *In sample (write -7 if unknown)*	Proportion (%) *	SD *	SE *	95% CI L *	95% CI U *	Statistical test * *For example: F, t, X2...*	Statistical test value *	p-value *
401	57.9	-7	-7	50.3	65.4	-7	-7	-7

Details on any **adjustment** carried out by authors, consider as well whether there is any **modelling** in the estimates of the effects. *
For example, for clustering.
The Proportion (%) of HepB timeliness assessed in 401Infants - children belonging to the group undefined, in time at end-line: 57.9 [SI

2 - Are you reporting data for participants: **Infants - children** on outcome **HepB timeliness** in group **HepB-UnIject • CHW** in time ? *
▸ more details
(●) Yes ( ) No

What statistic is used to summarise **HepB timeliness**? * 2
Note that if you are reporting an outcome in different ways (e.g. 'birth weight' as a continuous variable in Kg and as a proportion of participants above or below a certain threshold), then these should be considered as two different outcomes.
▸ more details
( ) Sum ( ) Count (●) Proportion (%) ( ) Other

N of **Infants - children** * *In sample (write -7 if unknown)*	Proportion (%) *	SD *	SE *	95% CI L *	95% CI U *	Statistical test * *For example: F, t, X2...*	Statistical test value *	p-value *
400	77.3	-7	-7	71.5	83	-7	-7	-7

Details on any **adjustment** carried out by authors, consider as well whether there is any **modelling** in the estimates of the effects. *
For example, for clustering.
The text mentions that clustering has been taken into account.

Are you reporting data for participants: **Infants - children** on *differences* of outcome **HepB timeliness** between **HepB amp. cold chain Hospital** and **HepB-UnIject • CHW** in time *
▸ more details
(●) Yes ( ) No

What statistic is used to assess the effects in **HepB timeliness**? * 3
Note that if you are reporting an outcome in different ways (e.g. 'birth weight' as a continuous variable in Kg and as a proportion of participants above or below a certain threshold), then these should be considered as two different outcomes.
▸ more details
(●) Proportions difference ( ) Absolute risk difference ( ) Hazard Ratio ( ) Risk Ratio ( ) Odds Ratio ( ) Other

OSSYPIS

N of **Infants - children** *In sample (write -7 if unknown)*	Proportions difference	SD	SE	95% CI L	95% CI U	Statistical test *For example: F, t, X2...*	Statistical test value	p-value
801	-7	-7	-7	-7	-7	X2	-7	p<0.0001

Details on any **adjustment** carried out by authors, consider as well whether there is any **modelling** in the estimates of the effects. *
For example, for clustering.

The Proportions difference of Antl-HBs titres after 3 doses (mIU/ml) assessed in 801Infants - children belonging to the group undefine

» 5·2·B - ROB 2 - Domain 2 (part 2) - Missing outcome data | HepB timeliness

[ROB2 - 2.6 - Adherence] Was an appropriate analysis used to estimate the effect of adhering to the intervention? *
Click here if you need help (will open a new window).
▸ *more details*

Yes

Copy and paste (or summarise) the narrative that supports your judgements. *
Use ['] (simple quotation marks) instead of ["] (double quotation marks), if needed. If pasting from PDF: manually remove line breaks, please.

The text reports on cold chain Issues and wastage due to overexposure to heat. suggesting the hospitals and community health workers Implemented the Intervention as expected.

The risk of bias in domain '**deviations from the intended interventions (adhering to intervention)**' is: '**Some concerns**'
If you do not agree with this assessment, state you assessment below and the reasons for disagreement.

» 5·2·B - ROB 2 - Domain 5 - Selection of the reported result | HepB timeliness

[ROB2 - 5.1] Were the data that produced this result analysed in accordance with a pre-specified analysis plan that was finalized before unblinded outcome data were available for analysis? *
Click here if you need help (will open a new window).
▸ *more details*

No information

[ROB2 - 5.2] Is the numerical result being assessed likely to have been selected, on the basis of the results, from multiple eligible outcome measurements (e.g. scales, definitions, time points) within the outcome domain? *
Click here if you need help (will open a new window).
▸ *more details*

Probably no

[ROB2 - 5.3] Is the numerical result being assessed likely to have been selected, on the basis of the results, from multiple eligible analyses of the data? *
Click here if you need help (will open a new window).
▸ *more details*

Probably no

Copy and paste (or summarise) the narrative that supports your judgements. *
Use ['] (simple quotation marks) instead of ["] (double quotation marks), if needed. If pasting from PDF: manually remove line breaks, please.

The outcome is well defined as consistent with standards. There is no much room to select results.

The risk of bias in domain '**measurement of the outcome**' is: '**Some concerns**'
If you do not agree with this assessment, state you assessment below and the reasons for disagreement.

» OVERALL RISK OF BIAS for HepB timeliness.

The overall judgment of the risk of bias for this outcome-comparison is **Some concerns**. If you disagree with this assessment, please, write an explanation below, or leave blank otherwise.
Disagreement may come from the fact that the study is judged to have some concerns for multiple domains in a way that substantially lowers confidence in the result.

→ Next

Back

COSSYRIS

KoboToolbox

CTC | SR 2 Data extraction

5 - Results | 1983_Wang_L._2007

» 5·3·A - AntI-HBs titres after 3 doses (mIU/ml) [in Infants - children]: HepB amp. outside CC - VHW vs HepB-UnIject • CHW | several points in time 3

Select the type of **data** that you are reporting here *
more details

Differences between two exposures or interventions, at a given point in time

Select the **participants** you want to report about *	Select the **interventions** to compare *	Outcomes *	Timing *
more details	more details	more details	more details
(•) Infants - children	[ ] HepB amp. cold chain Hospital<br>[✔] HepB amp. outside CC - VHW<br>[✔] HepB-UnIject • CHW	( ) HepB timeliness<br>(•) AntI-HBs titres after 3 doses (mIU/ml)	( ) at baseline<br>(•) at end-line

1 - Are you reporting data for participants: **Infants - children** on outcome **AntI-HBs titres after 3 doses (mIU/ml)** in group **HepB amp. outside CC - VHW** in time ? *
more details

(•) Yes ( ) No

What statistic is used to summarise **AntI-HBs titres after 3 doses (mIU/ml)**? * 1
Note that if you are reporting an outcome in different ways (e.g. 'birth weight' as a continuous variable in Kg and as a proportion of participants above or below a certain threshold), then these should be considered as two different outcomes.
more details

( ) Arithmetic mean ( ) Standardised mean ( ) Median p50
(•) Geometric mean

N of **Infants - children** * *In sample (write -7 if unknown)*	Geometric mean *	SD *	SE *	95% CI L *	95% CI U *	Statistical test * *For example: F, t, X2...*	Statistical test value *	p-value *
-7	93.3	-7	-7	-7	-7	-7	-7	-7

Details on any **adjustment** carried out by authors, consider as well whether there is any **modelling** in the estimates of the effects. *
For example, for clustering.
The outcome is well defined as consistent with standards. There is no much room to select results.

2 - Are you reporting data for participants: **Infants - children** on outcome **AntI-HBs titres after 3 doses (mIU/ml)** in group **HepB-UnIject • CHW** in time ? *
more details

(•) Yes ( ) No

What statistic is used to summarise **AntI-HBs titres after 3 doses (mIU/ml)**? * 2
Note that if you are reporting an outcome in different ways (e.g. 'birth weight' as a continuous variable in Kg and as a proportion of participants above or below a certain threshold), then these should be considered as two different outcomes.
more details

( ) Arithmetic mean ( ) Standardised mean ( ) Median p50
(•) Geometric mean

N of **Infants - children** * *In sample (write -7 if unknown)*	Geometric mean *	SD *	SE *	95% CI L *	95% CI U *	Statistical test * *For example: F, t, X2...*	Statistical test value *	p-value *
-7	102.3	-7	-7	-7	-7	-7	-7	-7

Details on any **adjustment** carried out by authors, consider as well whether there is any **modelling** in the estimates of the effects. *
For example, for clustering.
The outcome is well defined as consistent with standards. There is no much room to select results.

Are you reporting data for participants: **Infants - children** on *differences* of outcome **AntI-HBs titres after 3 doses (mIU/ml)** between **HepB amp. outside CC - VHW** and **HepB-UnIject • CHW** in time * 3
more details

KOSSRIS

◯ Yes ◉ No

» 5·3·B - ROB 2 - Domain 2 (part 2) - Missing outcome data | AntI-HBs titres after 3 doses (mIU/ml)

[ROB2 - 2.6 - Adherence] Was an appropriate analysis used to estimate the effect of adhering to the intervention? *
Click here *if you need help (will open a new window).*
▸ *more details*

Yes

Copy and paste (or summarise) the narrative that supports your judgements. *
Use ['] (simple quotation marks) instead of ["] (double quotation marks), if needed. If pasting from PDF: manually remove line breaks, please.

The text reports on cold chain issues and wastage due to overexposure to heat, suggesting the hospitals and community health workers implemented the intervention as expected.

The risk of bias in domain '**deviations from the intended interventions (adhering to intervention)**' is: '**Some concerns**'
If you do not agree with this assessment, state you assessment below and the reasons for disagreement.

» 5·3·B - ROB 2 - Domain 5 - Selection of the reported result | AntI-HBs titres after 3 doses (mIU/ml)

[ROB2 - 5.1] Were the data that produced this result analysed in accordance with a pre-specified analysis plan that was finalized before unblinded outcome data were available for analysis? *
Click here *if you need help (will open a new window).*
▸ *more details*

No information

[ROB2 - 5.2] Is the numerical result being assessed likely to have been selected, on the basis of the results, from multiple eligible outcome measurements (e.g. scales, definitions, time points) within the outcome domain? *
Click here *if you need help (will open a new window).*
▸ *more details*

Probably no

[ROB2 - 5.3] Is the numerical result being assessed likely to have been selected, on the basis of the results, from multiple eligible analyses of the data? *
Click here *if you need help (will open a new window).*
▸ *more details*

Probably no

Copy and paste (or summarise) the narrative that supports your judgements. *
Use ['] (simple quotation marks) instead of ["] (double quotation marks), if needed. If pasting from PDF: manually remove line breaks, please.

We have no rea sons to think that laboratory test results have been selected in any way.

The risk of bias in domain '**measurement of the outcome**' is: '**Some concerns**'
If you do not agree with this assessment, state you assessment below and the reasons for disagreement.

» OVERALL RISK OF BIAS for AntI-HBs titres after 3 doses (mIU/ml).

The overall judgment of the risk of bias for this outcome-comparison is **Some concerns**. If you disagree with this assessment, please, write an explanation below, or leave blank otherwise.
Disagreement may come from the fact that the study is judged to have some concerns for multiple domains in a way that substantially lowers confidence in the result.

➔ Next

Back

COSSYRIS

KoboToolbox

CTC | SR 2 Data extraction

6 - Study conclusion | 1983_Wang_L._2007

Copy and paste (or summarise) the conclusion of the whole study, **as stated by the authors**. *

This village-based, out-of-cold chain strategy Is relevant to the global reduction of HBV Infection, since WHO estimates that more than 50% of the world's children are born at home.1 This strategy could be applied In many countries where health workers live among populations that are not accessible by the formal health system. It could be beneficial not only in geographically isolated areas, but also in urban areas where cultural differences impede access to clinic-based immunizations.

Use this space to annotate any observation in relation to the contents or functionality of the form. Your observations will be read and processed during the next stage of the review. No other observations will be taken into account.

Infants were recruited at birth. The end-line survey was carried out selecting infants 1 to 11 months of age. There is also baseline data, although it has not been used to estimate the effects of the intervention.

Consider whether you have to collect information about the corresponding author of 1983_Wang_L._2007.

Family name	First name	Email

Submit and the next reference will appear. Thank you very much for your time and contributions.

You cannot submit the form because its functionality is linked to a user account.

Submit

Back

Return to Beginning

Go to End

OSSYRIS
